# The Morphology of the Rare Beetle *Silphopsyllus desmanae* (Leiodidae), a Commensal of the Semiaquatic Russian Desman

**DOI:** 10.1002/jmor.70031

**Published:** 2025-02-26

**Authors:** Paweł Jałoszyński, Odair M. Meira, Margarita I. Yavorskaya, Alexandr Prokin, Veit Grabe, Rolf G. Beutel

**Affiliations:** ^1^ Museum of Natural History University of Wrocław Wrocław Poland; ^2^ Institut für Zoologie und Evolutionsforschung Friedrich‐Schiller‐Universität Jena Jena Germany; ^3^ Laboratório de Biologia Comparada e Abelhas (LBCA), Departamento de Biologia FFCLRP Universidade de São Paulo Ribeirão Preto Brazil; ^4^ Institut für Evolution und Ökologie Universität Tübingen Tübingen Germany; ^5^ Papanin Institute for Biology of Inland Waters, Russian Academy of Sciences Borok Russia; ^6^ Max‐Planck‐Institut für Chemische Ökologie Jena Germany

**Keywords:** anatomy, commensalism, musculature, phylogeny, Platypsyllinae, skeleton

## Abstract

*Silphopsyllus desmanae*, a species of the small subfamily Platypsyllinae of Leiodidae, lives in the fur of the semiaquatic Russian desman, and is apparently adapted to this highly specialized life style. Even though the morphology of adults of the species was described almost 70 years ago, we re‐examined it with modern methods and documented its external and internal features in detail, and discuss them with respect to phylogeny and function. Our analyses of morphological data place *Leptinillus* as the sister group of the remaining genera of Platypsyllinae, and *Leptinus* as the sister group of *Silphopsyllus* + *Platypsyllus*. Platypsyllinae are supported by many putative autapomorphies: supraantennal ridges directed mesad but not extending beyond the antennal insertions and not forming a transverse ridge; tentorium without connected laminatentoria anterior to the tentorial bridge; submentum subrectangular; labrum about as wide as the maxillary‐labial complex; elongate and posteriorly projecting lateral lobes of the mentum; antennomeres lacking periarticular gutters (and Hamann's organs); cervical sclerites absent; precoxal prosternal region distinctly longer than the coxal rests; mesocoxal cavities situated closer to the body midline than to the lateral mesothoracic margins; metanepisterna fused with the metaventrite; metascutum laterally overlapping the meso‐ and metapleural regions; procoxae subglobose or only slightly elongate; mesocoxae subglobose. Platypsyllinae are most likely the sister group of Coloninae + Cholevinae. Eight unique apomorphies differentiating *Platypsyllus* from all the remaining Platypsyllinae are mainly adaptations for living in the fur of beavers. *Silphopsyllus* is much less adapted to life on a semiaquatic host than *Platypsyllus*.

## Graphical abstract

We document in detail the morphology of *Silphopsyllus desmanae* (Leiodidae), a commensal of the endangered semiaquatic Russian desman, and discuss it with respect to phylogeny and function. *Silphopsyllus* is much less adapted to life on a semiaquatic host than the closely related beaver beetle *Platypsyllus*. Our analyses of morphological characters place *Leptinillus* as sister to the remaining Platypsyllinae, and Platypsyllinae as sister to (Coloninae + Cholevinae).

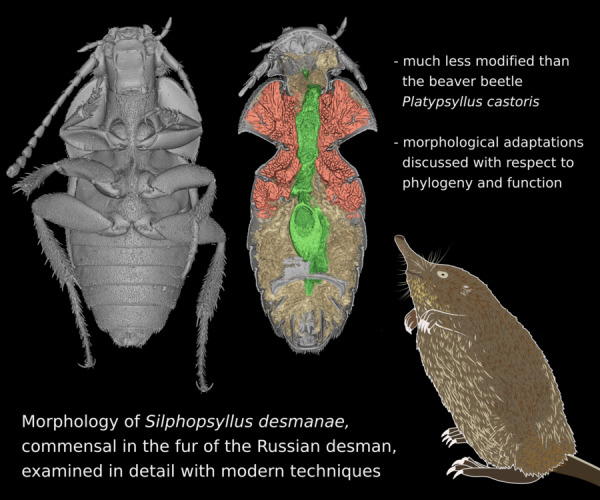

## 1 Introduction

*Silphopsyllus desmanae* Olsufiev is a rarely collected beetle species of the small subfamily Platypsyllinae of Leiodidae (Staphylinoidea). It lives in the fur of the semiaquatic Russian desman (*Desmana moschata* (Linnaeus)), the ‘vykhukhol’, and has a wide distribution area in Central European and South European Russia, in the basins of the Don, Volga, and Ural River (Olsufiev 1923). That the hosts also occur in Ukraine and Kazakhstan (Kennerley and Turvey 2016) suggests the occurrence of the beetle in these countries, but this requires confirmation. The adult beetles were described by G. Olsufiev based of materials collected in Sura River, Penza Oblast (Olsufiev 1923), and the larvae few years later by Semenov‐Tian‐Shansky and Dobzhansky (1927), using material collected at Matyra River in Lipetsk Oblast.

The host of *Silphopsyllus desmanae*, the Russian desman, lives at the edges of slow flowing rivers and ponds. However, details of the biology of the beetles are presently not available. It is assumed that they feed on moist masses of epidermis, tissues and liquid of the corium, lymph, and possibly blood seeping from small capillary vessels (Pavlovsky 1956).

In this study, we present comprehensive locality data for *Silphopsyllus desmanae* and also observations on its biology. The adult morphology was already described by Pavlovsky (1956), using light microscopy and also histology, including some internal features, especially of the head. However, a recent anatomical study on the closely related beaver beetle *Platypsyllus castoris* Ritsema (Yavorskaya et al. 2023) inspired us to reinvestigate the vykhukhol’ beetle with different techniques, notably micro‐computed tomography (µ‐CT), computer‐based 3D reconstruction, and SEM. Due to the suboptimal fixation of the available specimens, the main focus is on external and internal skeletal structures. However, short statements are also provided on internal soft parts (e.g., muscles, central nervous system, digestive tract) when features of these elements were sufficiently preserved. The state of preservation is visible in renders shown in Figure 7. The observed features are discussed in the context of characters recently presented in the study on *Platypsyllus castoris*, with respect to functional, phylogenetic and evolutionary implications. In contrast to Yavorskaya et al. (2023) we use a more extensive morphological data set, mainly to infer the systematic placement of the specialized species of Platypsyllinae in the family Leiodidae.

## 2 Materials and Methods

### 2.1 Studied Species

Specimens of *Silphopsyllus desmanae* provided by Alexandr Prokin (see 3.1.1.) were fixed in 70% ethanol. Internal softparts such as for instance muscles or glands were only preserved partially. The preservation is better in the postcephalic body than in the head (Figure 7). Specimens of *Leptinillus validus* fixed in 70% ethanol were obtained from the Field Museum of Natural History (Chicago, Ill. U.S.A.).

The classification of Leiodidae we use follow that of the Catalogue of Life (Bánki et al. 2024).

### 2.2 Micro‐Computed Tomography (µCT)

The specimen was dehydrated in an ascending series of ethanol (80%, 90%, 96%, 100%), and then stained for contrast in an iodine solution (1% iodine in 100% ethanol) for 3 days. After this, it was transferred to 100% ethanol and placed in the distal portion of a pipette tip filled with the same liquid. The upper end was closed with Patafix (UHU, Bühl, Germany), the lower end by melting. The sample was then scanned with a stable energy beam of 18 keV, 0.5 µm pixel size at the Deutsches Elektronen Synchrotron (DESY; beamlines DORIS III/BW2 and PETRA III/IBL P05, operated by the Helmholtz‐Zentrum Geesthacht, Hamburg, Germany).

### 2.3 3D Reconstruction and Image Processing

The obtained data were cropped and sampled using Dragonfly v. 2020.1.0.797 (Object Research Systems, Montreal, Quebec, Canada). For creating the .tif stacks we used Amira macro “Multi Export” (Engelkes et al. 2018). Amira v. 6.0.1 and 6.1.1 (Thermo Fisher Scientific, Hillsboro, OR) and 3D Slicer 5.6.2 were used for the segmentation. Selected structures (e.g., tentorium, nervous system, musculature) were presegmented manually in 3D Slicer and subsequently semi‐automatically segmented using Biomedisa (Lösel et al. 2020). “Mask Volume” tool in 3D slicer was used to substract the volume for the selected structures. Finally, the “Slicermorph” extension for 3D Slicer was used for obtaining high resolution screenshots of the combined (surface + volume) rendering of all the visualized structures.

### 2.4 Scanning Electron Microscopy (SEM)

One specimen each of *Silphopsyllus desmanae* and *Leptinillus validus* were cleaned in a 10% aqueous solution of KOH for 12 h, washed in glacial acetic acid and distilled water (Schneeberg et al. 2017), dehydrated in an ascending ethanol series and critically‐point dried with Polaron 3100. The beetles were coated with gold (EmitechK 500X), mounted on a rotatable specimen holder and images of the habitus were taken with a SEM Zeiss EVO LS 10. The same specimens were then dissected and isolated body parts were mounted both on stubs and on the rotatable holder, coated with gold and examined with the SEM.

### 2.5 Image Plates

GIMP 3.0.0 (GPL license) was used to edit the micrographs. Inkscape 1.3 (GPL license) was used for preparation of the digital drawings and for arranging and labeling the figure plates.

### 2.6 Cladistic Analysis

The phylogenetic analysis was based on 105 (numbered from 0) non‐additive and unordered adult exoskeletal characters; inapplicable character states were assigned a gap value (“–“) and treated equivalent to missing data (“?”). The character states were scored for 24 genera of Leiodidae. As outgroup we chose the genus *Agyrtes* Froelich of Agyrtidae, the well‐established sister group of Leiodidae (e.g., McKenna et al. 2015). The characters and character states are listed in Supporting online material Appendix S3 (additional images are shown in S1 and S2). The data matrix was assembled in Nexus Data Editor for Windows v. 0.5.0 (Page 2001) (Appendix S4). Parsimony analyses were conducted with TNT v.1.5 (Goloboff et al. 2008) applying the “traditional search” strategy, and also using implied weighting (weighting function K = 6). The tree bisection reconnection swapping algorithm was applied, with 1000 replicates and 1000 trees saved per replication. The standard bootstrap values were also calculated in TNT (1000 repl.), and character mapping was performed in WinClada v. 1.00.08 (Nixon 1999–2002). Trees were exported from TNT and annotated in CorelDraw Graphic Suite 2017.

### 2.7 Terminology

Like in the study on the beaver beetle (Yavorskaya et al. 2023), the muscles of the head were named following the terminology of v. Kéler (1963), and those of the thorax Larsén (1966). Beutel et al. (2014) was used for general morphological terminology.

## 3 Results

### 3.1 Distribution Record and Biological Data

#### 3.1.1 Distribution

The type material used by Olsufiev (1923) was collected at Sura River, Penza Province (≈ N53.0908, E45.5588) by the author of that study, and by S.P. Korovin and S.V. Dyukin. Specimens were also reported (pers. comm. of O. I. Semenov‐Tian‐Shansky) from Matyra River ( ≈ N52.48, E40.05) in Lipetsk District of Tambov Oblast (now Lipetsk Oblast) (Olsufiev 1923). The immature stages used for the larval description by Semenov‐Tian‐Shansky and Dobzhansky (1927) were collected at the Matyra river locality. The species was also recorded for the Voronezh Oblast from Khoperskiy Nature Reserve ( ≈ N51.21, 41.69) and the railway station Somovo ( ≈ N51.75, E39.34) in the Usman pine‐forest (Barabash‐Nikiforov 1950). Recently, a number of specimens were collected during an investigation on the biology of the Russian desman in the Lipetsk Oblast by Alexander I. Zemlyanukhin (Prokin and Zemlyanukhin 2008; Zemlyanukhin 2009).

The documented Russian distribution area of *Silphopsyllus desmanae* comprises the Penza, Lipetsk and Voronezh regions [Oblast] (Olsufiev 1923; Semenov‐Tian‐Shansky and Dobzhansky 1927; Barabash‐Nikiforov 1950; Prokin and Zemlyanukhin 2008; Zemlyanukhin 2009). All findings of recent years are from Lipetsk Oblast only. In this region *Desmana moschata* is known only from the Oka‐Don Lowland, where the mammals prefer small slowly flowing rivers and small floodplane lakes (Zemlyanukhin 2009). Voronezh records (Barabash‐Nikiforov 1950) are also from Oka‐Don Lowland. No records are known from the Middle Russian Upland, a more elevated higher part of Voronezh and Lipetsk Regions, where rivers are characterized by faster water flow.

Recently studied material is listed in the following:

− N52.334376, E40.383401: Lipetsk Oblast, Dobrinskyi District, env. village Veselovka, Plavitsa River, 27.03.2007, 6 ♂♂, 3 ♀♀, leg. A.I. Zemlyanukhin, from adult male of *D. moschata*; 29.03.2008, 27 ♂♂, 30 ♀♀, leg. A.I. Zemlyanukhin, from adult male of *D. moschata*.
− N52.218459, E40.640037: Lipetsk Oblast, Dobrinskyi District, env. village Novopetrovka, Chamlyk River, 29.03.2008, 6 ♂♂, 8 ♀♀, 1 larva, leg. A.I. Zemlyanukhin, from adult male of *D. moschata*; 12.07.2008, 1 ♂, leg. A.I. Zemlyanukhin, from adult male of *D. moschata*.
− N52.825154, E39.753393: Lipetsk Oblast, Dobrovskyi District, env. vill. Panino, Martynchik River, 10.03.2007, 1 ♂, leg. A.I. Zemlyanukhin, from adult male of *D. moschata.*

These materials were identified by A. Prokin. The larva was compared with historical specimens (Semenov‐Tian‐Shansky and Dobzhansky 1927), kept in the Zoological Institute of the Russian Aсademy of Sciences, St. Petersburg (A.G. Kirejtshuk) and in the collection of the Moscow State Pedagogical University (A.A. Zaitsev).

#### 3.1.2 Biology

As reported by Zemlyanukhin (2009) the number of beetles found on hosts is relatively low. Only five out of a total of 34 examined specimens ( = 14.7%) of *Desmana moschata* yielded *Silphopsyllus desmanae*, but 57 exemplars were observed on one host (average = 17 individuals/host). The sex ratio was close to 1:1. It appears likely that *S. desmanae* is another commensal species of Platypsyllinae, like *P. castoris* from the same subfamily (Yavorskaya et al. 2023). Most of the adult beetles were collected in spring, which may be due to dispersal (settling) on migrating adult males of *D. moschata* (Zemlyanukhin 2009). Larvae were only collected on two occasions, 02.07.1926 (Semenov‐Tian‐Shansky and Dobzhansky 1927) and 29.03.2008 (Zemlyanukhin 2009).

### 3.2 Morphology of Adults

#### 3.2.1 General Features

**FIGURE 1:**
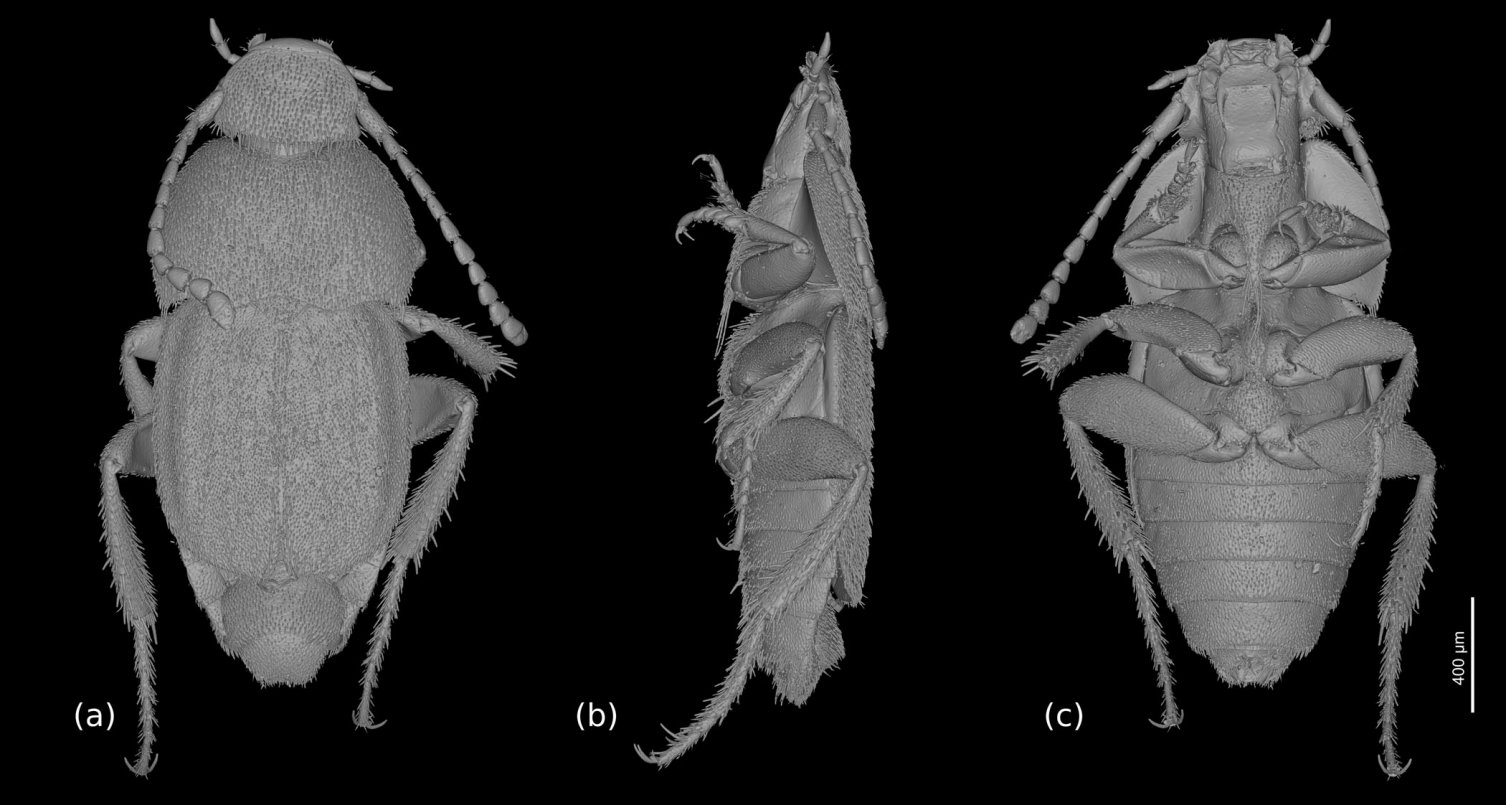
*Silphopsyllus desmanae*, 3D reconstructions of the habitus. (a) dorsal view; (b) lateral view; (c) ventral view.

The beetles are flattened, approximately oval in dorsal view, with the elytral apex truncated (Figure 1a) and the elytra widening posteriorly in females. The body is ca. 3.3 mm long and the maximum width at the posterior pronotal corner and posterior elytral margin is ca. 1.7 mm. The coloration of the cuticle is light brown in almost all areas; almost the entire body surface is covered with a dense vestiture of short setae (ca. 50 µm); additionally, clusters of longer and thicker setae (ca. 0.1 mm) are present in some areas. The walking legs are well developed and the hind legs are distinctly longer than the other two pairs; a row of spines is present on the outer edge of the tibiae; the paired symmetrical claws are well developed (Figure 1b, c).

#### 3.2.2 Head

##### 3.2.2.1 External Head Capsule

**FIGURE 2:**
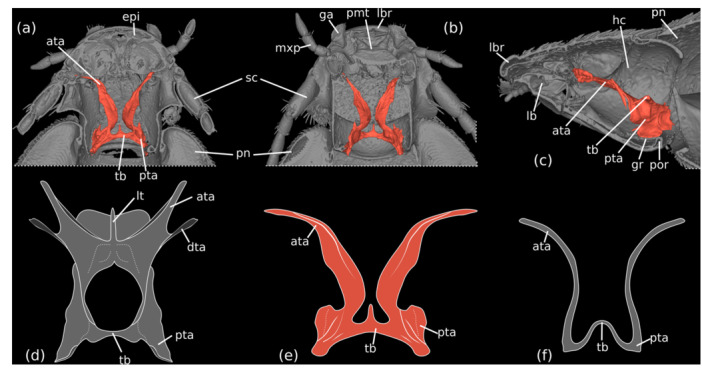
*Silphopsyllus desmanae*, 3D reconstructions of external and internal cephalic structures (a–c) and schematic drawings of tentorial structures in different leiodids in dorsal view (d–f). (a) horizontal section, dorsal view; (b) horizontal section, ventral view; (c) sagittal view; (d) *Catops ventricosus* (Cholevinae; based on 3D reconstruction from Antunes‐Carvalho et al. 2017a); (e) *Silphopsyllus desmanae* (Platypsyllinae); (f) *Platypsyllus castoris* (Platypsyllinae; based on 3D reconstruction from Yavorskaya et al. 2023). ata, anterial tentorial arm; dta, dorsal tentorial arm; epi, epipharynx; ga, galea; gr, gular ridge; hc, head capsule; lb, labium; lbr, labrum; lt, laminatentoria; mxp, maxillary palp; pmt, prementum; pn, pronotum, por, postoccipital ridge; pta, posterior tentorial arms; tb, tentorial bridge.

**FIGURE 3:**
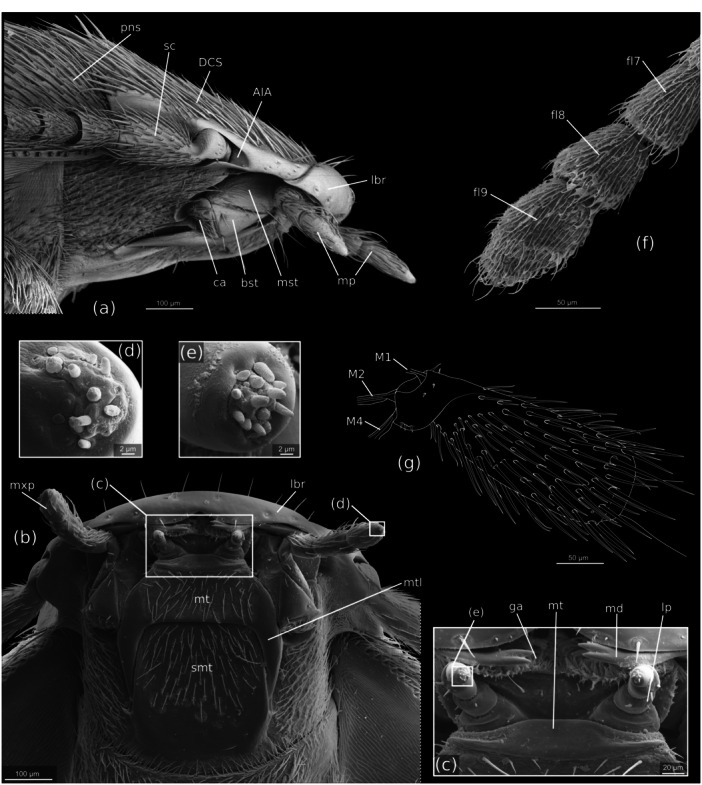
*Silphopsyllus desmanae*, SEM micrographs of the cephalic structures and a schematic drawing of the antennal base (g). (a) head, lateral view; (b) head, ventral view; (c) parts of mouthparts, ventral view; (d) distal end of apical maxillary palpomere; (e), distal end of apical labial palpomere; (f) distal flagellomeres of the right antenna, ventral view. AIA, antennal insertion area; bst, basistipes; ca, cardo; DCS, dorsal cephalic shield; fl7/8/9, antennal flagellomeres 7/8/9; ga, galea; lbp, labial palp; lbr, labrum; lp, labial palp; md, mandible; mp, maxillary palp; mt, mentum; mtl, mental lobes; mxp, maxillary palp; M1, *M. tentorioscapalis anterior*; M2, *M. tentorioscapalis posterior*; M4, *M. tentorioscapalis medialis*; pns, pronotal shield; smt, submentum.

The head is prognathous, only slightly inclined, and distinctly retracted posteriorly, with the posterodorsal part concealed by the anterior pronotum. It is moderately flattened and appears wedge‐shaped in lateral view (Figure 1b). The thickness of the cuticle is not increased. The shield‐like exposed dorsal side of the head capsule (DCS = dorsal cephalic shield) appears approximately bell‐shaped, slightly wider than long; it is anteriorly nearly straight, slightly convex, but its anterolateral corners are broadly rounded. The posterior corners of the DCS are also rounded. The narrow occipital region is retracted into the prothorax; it is partially covered by the posterodorsal cephalic projection (PCP = posterodorsal cephalic projection) of the DCS, which is laterally adjacent with the anterior pronotal margin; its posterior margin is slightly convex; it bears a row of longer setae, but lacks regularly arranged broad and flattened setae. A broadly rounded posteromedian projection (PMCP = posteromedian cephalic projection) is present posterior to the PCP, shaped like a transverse scutellar shield; the surface is smooth and a pair of setae is inserted close to the midline; this structure is exposed in the anteromedian emargination of the pronotum. Ecdysial lines, that is, the frontal and coronal sutures, are absent on the flat dorsal side of the head capsule. The DCS bears a dense, regular vestiture of short setae (ca. 50 µm) with their sockets placed in shallow concavities. The internal clypeofrontal strengthening ridge is present and in ethanol‐preserved specimens visible through the cuticle as a dark line on each lateral 1/3 of the clypeal width; externally this incomplete ridge is not marked by an inflection but by a weakly elevated ridge, poorly visible in SEM images, but discernible using a stereomicroscope at various lighting angles and in sagittal 3D renderings (Figure 2c). A transverse, evenly rounded anteromedian sclerite, distinctly separated from the main portion of the head capsule by a complete and deep transverse furrow, is in fact a strongly modified, enlarged and immobilized labrum (AMLS=anteromedian labral shield). The antennal insertion area (AIA) is located laterally in the middle region of the DCS; the globular basal articulatory piece of the scapus is visible from above; like the AMS, the areas below and posterior to the AIA have a smooth, glabrous surface; a rim for reception of the proximal shaft of the scapus is present on the DCS posterolaterally; ventrally it is delimited by a sharp edge (Figure 3a). The roughly triangular and fairly large genal region is delimited by this edge on the dorsal side and by the gula and submentum ventrally; it is narrowing anteriorly towards the insertion area of the paired mouthparts and widening posteriorly; on most of its surface it bears a vestiture of setae similar to those of the DCS; its posteriormost portion is glabrous. The exposed posterior part of the ventral head capsule is parallel‐sided in dorsal view, almost cylindrical, and about 2/3 as wide as the maximum width of the DCS at its posterior edge; its surface bears a regular dense vestiture of short setae, except for a fairly large posteromedian submental region. A moderately sized, rounded and shallow maxillary groove is present laterad the anterolateral submental margin. The gular region is retracted into the prothorax, covered by the prosternum (Figure 2a–c).

##### 3.2.2.2 Cephalic Endoskeletal Structures

The cephalic endoskeleton is normally developed; vertical cuticular columns (see Yavorskaya et al. 2023) and novel reinforcement ridges are missing (Figure 2a–c, e). The short but strongly developed posterior tentorial arms are fused with short, oblique gular ridges, which are laterally continuous with the well‐developed postoccipital ridge; the upper edges of the ridges are connected by a robust, nearly straight tentorial bridge. A well‐developed straight anteromedian projection of the tentorial bridge is present (Figure 2a, b). The dorsal arms are vestigial or absent. The strongly developed, long and slightly curved anterior arms extend anterolaterad and are connected to the head capsule at the posterolateral margin of the clypeal region; the anterior tentorial grooves are not visible externally. A medially continuous laminatentorium in front of the tentorial bridge is not present.

##### 3.2.2.3 Labrum

**FIGURE 4:**
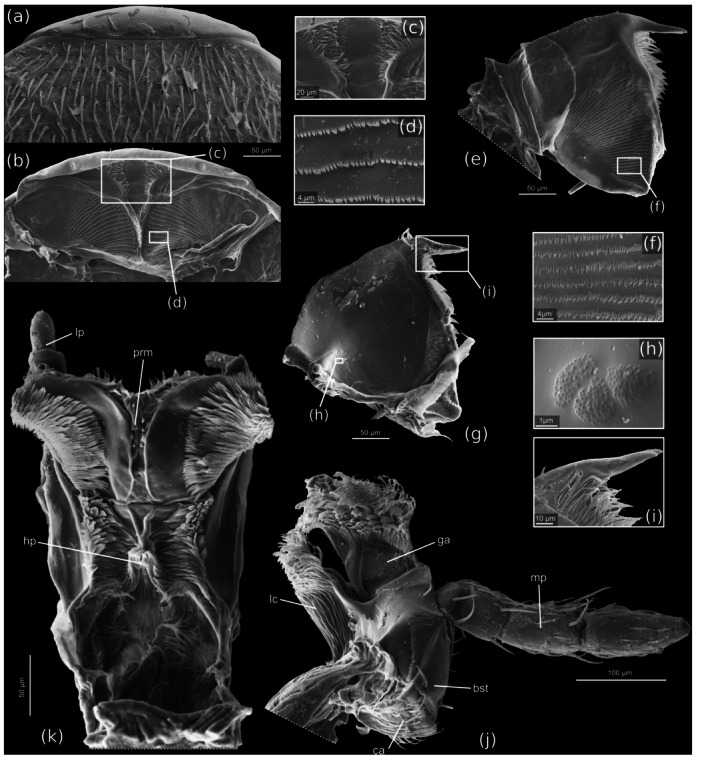
*Silphopsyllus desmanae*, scanning electron micrographs of dissected mouthparts. (a) labrum and fragment of dorsal cephalic shield, dorsal view; (b) anterior epipharynx, ventral view; (c) anteriormost epipharyngeal section; (d) spine rows on lateral lobes of anterior epipharynx; (e) left mandible, dorsal view; (f) spine rows on dorsal mandibular surface; (g) right mandible, ventral view; (h) cuticular sculpture on ventral side of mandible; (i) apical tooth of the right mandible, ventral view; (j) right maxilla, dorsal view; (k) labium, dorsal view. bst, basistipes; ca, cardo; ga, galea; hp, anterior hypopharynx; lc, lacinia; lp, labial palp; mp, maxillary palp; prm, prementum.

The labrum is transformed into a completely immobilized anteromedian labral shield (AMLS), which is fully exposed; a complete and distinct transverse furrow separates it from the clypeal region; an internal connecting membrane is vestigial or lacking. A regular row of 8–10 longer setae is present close to its hind margin, but otherwise the surface is largely smooth; two pairs of minute sensilla are inserted on the anterolateral region and some short setae close to the anterior margin (Figure 4a, b). The anterior edge displays a distinct median emargination and appears thus bilobed. The structure designated as anteromedian sclerite (AMS) of *Platypsyllus* (Yavorskaya et al. 2023) is in fact a distinctly modified, immobilized labrum. Posterolateral tormae are not recognizable and apparently absent.

##### 3.2.2.4 Epipharynx

The anterior epipharynx, that is, the ventral labral wall and the roof of the laterally open cibarium, is situated close to the dorsal wall of the head capsule; anteromesal bulges, arranged in a V‐shaped manner and further dividing into two branches anteriorly, divide the anteriormost epipharyngeal section into two equal halves; the bulges bear medium‐length and short microtrichia. The lateral halves of the anterior epipharynx are covered with dense parallel rows of minute spines, similar to those on the mandibular dorsal surface (Figure 4b–d). The posterior epipharynx is laterally fused with the posterior hypopharynx; together they form a short, laterally closed prepharyngeal tube.

##### 3.2.2.5 Antennae

In dorsal view the basal articulation of the long and 11‐segmented antennae is slightly exposed by a shallow lateral indentation of the dorsal cephalic shield (DCS); it is freely exposed in lateral view but covered by a rounded and smooth flat lobe on the ventral side (Figure 3a, b). The antenna is of the filiform type, only slightly widening in its distal half; it reaches the metathoracic‐abdominal border region posteriorly. The entire antennal surface is covered with a dense vestiture of short and medium length setae, the longer ones mainly inserted laterally and distally. The scapus is distinctly longer than the other antennomeres, approximately twice as long as the pedicellus and the proximal flagellomeres; it is composed of a fairly large, globular articulatory piece and a much longer but narrower cylindrical distal portion (Figure 3g). The pedicellus is about half as long as the total length of the scapus and slightly thinner. The following five antennomeres are similar in size and shape, only slightly narrower. The distal four antennomeres are slightly shorter, somewhat flattened, and slightly widening towards their apex; they display a slightly exposed basal peduncle; antennomere 9 is slightly longer than the two neighboring segments; the apical antennomere is spindle‐shaped and longer than the preceding ones and its distal region is abruptly flattened dorsoventrally (Figure 3f).

##### 3.2.2.6 Mandibles

The distinctly flattened mandibles are articulated in a typical dicondylic manner, with a distinct condyle of the primary mandibular joint on its ventral basal margin. The shape is approximately triangular, with a distinctly rounded lateral and mesal margin and a single, mesally directed slender apical tooth with a bifurcate apex (Figure 4g, i). The dorsal surface of the mandible is weakly convex, and the ventral surface weakly impressed. The mesal margin proximad the apical tooth is densely covered with long microtrichia which almost reach the proximal 1/3 of the mandibles. A molar region is present proximally and densely covered with asperities. A large dorsal area adjacent to the mesal margin and expanding to about half of the mandibular width is covered with parallel rows of densely arranged minute spines similar to those of the anterolateral epipharynx and apparently interacting with them in the processing of food (Figure 4e, f). A flat and glabrous proximolateral mandibular region is separated from the remaining dorsal surface by a distinct rounded edge.

##### 3.2.2.7 Maxillae

The maxillae (Figure 4j) are inserted in the shallow maxillary grooves. The cardo is small, transverse, and its exposed surface and the lateral side are covered with short setae. The stipes is composed of a narrow triangular basistipes and a mediostipes, which is mesally fused with the lacinia; the former has a smooth surface and bears only few setae; the latter is largely glabrous and partly covered by the mentum in the resting position of the maxilla. Laterally an elongate palpifer is connected with the lateral margin of the basistipes; a long and thick seta is inserted on it distally, and several shorter and thinner setae on its outer surface. The small, curved palpomere 1 is apically inserted on the palpifer; it bears a single seta; palpomere 2 is distinctly larger, slightly longer than half of the palpifer, and cylindrical; palpomere 3 is slightly longer and slightly clavate; the apical palpomere is spindle‐shaped, and about twice as long as the second segment; all palpomeres bear a vestiture of short setae and a group of minute sensilla is present on the apical region of the ultimate segment (Figure 3d). The well‐developed lacinia is distally densely covered with apically curved and spatulate microtrichia and a row of microtrichia is inserted along its mesal edge. The galea is composed of a distinctly sclerotized trapezoidal basigalea with a smooth, glabrous surface, and of a distigalea which appears less sclerotized; the distigalea is distally densely covered with curved microtrichia, similar to those of the distal lacinia (Figure 4j).

##### 3.2.2.8 Labium

The large submentum is subquadrate and laterally enclosed by the posterolateral projections of the mentum; posteriorly it is fused with the gula, but it is distinctly demarcated from the genal area by lateral submental sulci, which are accentuated by the elevated position of the submentum in relation to the genae. The surface of the submentum is smooth, and the posterior region glabrous; the rest bears a moderately dense vestiture of medium length adpressed setae. The lateral edges of the submentum are slightly rounded and slightly converging anteriorly; the anterolateral corners are rounded and the anterior margin straight. The mentum is well developed and clearly separated from the submentum. A conspicuous feature is the presence of a pair of posterolateral projections, narrowing posteriorly and apically pointed, with a smooth and glabrous surface; they reach the posterior third of the submentum (Figure 3b). The posterior edge of the mentum between the projections is almost straight, only slightly concave. The surface of the anterior main part of the mentum bears a vestiture of setae similar to that of the anterior submental portion. The short prementum is fused with the straight anterior edge of the mentum, but the border is still recognizable. A short, smooth and sclerotized stripe is followed by an equally short membranous region with a broad slightly projecting median portion. The short palps are inserted anterolaterally on the membranous part of the prementum; the cylindrical palpomere 1 is short and broad, about twice as wide as the equally short and cylindrical palpomere 2; the apical palpomere 3 is about twice as long as 2, cylindrical and apically rounded, with a group of minute sensilla‐like pegs on its apex (Figure 4k).

##### 3.2.2.9 Hypopharynx

The anterior hypopharynx is fused with prelabium, both forming a compact structural unit that appears trapezoidal in cross section. Two semicircular, moderately sclerotized lobes are present above the prementum, with rounded mesally directed margins, and laterally with dense brushes formed by several densely arranged rows of microtrichia; the lobes are medially separated by ca. 20 µm. A middle region of the hypopharynx is separated from the anteriormost section by a distinct, deep transverse furrow; this area is also equipped with dense microtrichial brushes, both mesally bordered by bulges which are almost adjacent medially (Figure 4k). The posterior hypopharynx forms the floor of the prepharyngeal tube.

##### 3.2.2.10 Pharynx

The pharynx, posteriorly adjacent with the prepharyngeal tube, is fairly wide and appears approximately round in cross section on the µ‐CT scans.

##### 3.2.2.11 Cephalic Musculature

The cephalic musculature is largely decayed; only vestiges of few muscles are preserved, likely of the mandibles (especially *M. craniomandibularis internus*, M.11) and maxillae (extrinsic muscles, likely *M. tentoriocardinalis* and *M. tentoriostipitalis*, M.17, M.18). Insertion points of three antennal muscles M.1, M.2 and M.4 (*M. tentorioscapalis anterior*, *M. tentorioscapalis posterior* and *M. tentorioscapalis medialis*) and vestiges of their tendons were visible in SEM micrographs (Figure 3g).

##### 3.2.2.12 Cephalic Nervous System

The brain is located in the posterodorsal cephalic region; it is not conspicuously enlarged in relation to the size of the head; optic lobes are not developed. The suboesophageal ganglion is located in the posteroventral area of the head, partly enclosed by the short gular ridges, the posterior tentorial arms, and the tentorial bridge (Figure 7b).

##### 3.2.2.13 Cephalic Glands

Not clearly recognizable due to insufficient preservation.

#### 3.2.3 Prothorax

**FIGURE 5:**
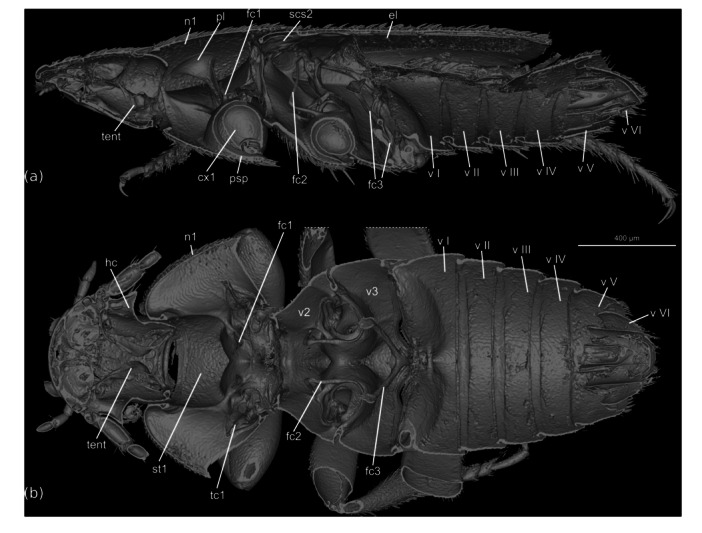
*Silphopsyllus desmanae*, 3D reconstructions of external and internal cuticular structures. (a) sagittal view; (b) horizontal section, dorsal view. cx1, procoxa; el, elytra; fc1/2/3, pro‐/meso‐/metafurca; hc, head capsule; n1, pronotum; psp, prosternal process; scs2, mesoscutellar shield; pl, propleura; st1, prosternum; tc1, protrochantin; tent, tentorium; v2/3, meso‐/metaventrite.

**FIGURE 6:**
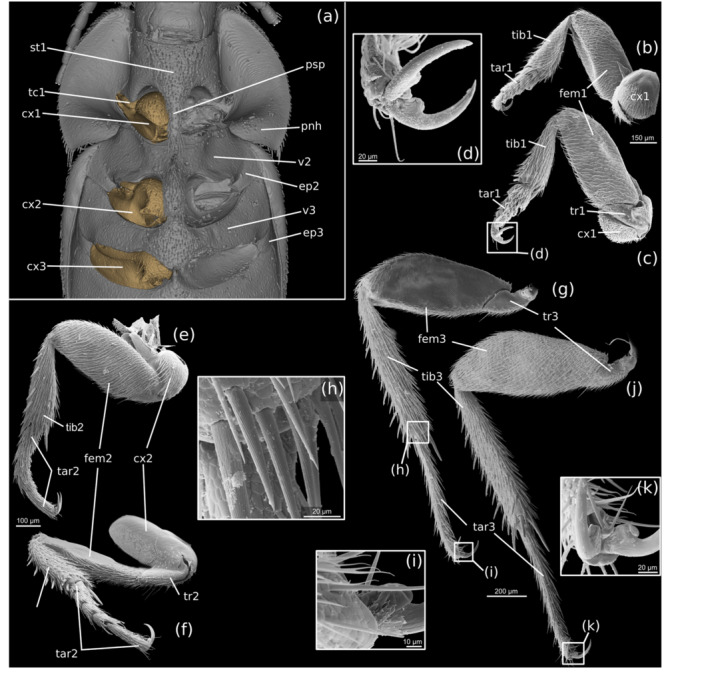
*Silphopsyllus desmanae*, 3D reconstruction and SEM micrographs of thoracic structures. (a) thorax, ventral view, leg elements distad the coxa removed; (b) fore leg, dorsal view; (c) fore leg, ventral view, (d) fore leg, claws; (e) middle leg, dorsal view; (f) middle leg, ventral view; (g) hind leg, dorsal view; (h) spines on apical region of metatibia; (i) apex of metatarsus; (j) hind leg, ventral view; (k) apex of metatarsus, ventral view. cx1/2/3, pro‐, meso‐, metacoxa; ep2/3, mes‐/metepimeron; fem1/2/3, pro‐/meso‐/metafemur; pnh, pronotal hypomera; psp, prosternal process, st1, prosternum; tar1/2/3, pro‐/meso‐/metatarsus; tc1, protrochantin; tib1/2/3, pro‐/meso‐/metatibia; tr1/2/3, pro‐/meso‐/metatrochanter; v2/3, meso‐/metaventrite.

The prothorax on its dorsal side is about 1/3 as long as the entire body (Figure 1a). The shield‐like pronotum is moderately arched and about twice as wide at its hind margin as the length in midline; it is densely covered with adpressed setae, mostly short, but longer ones are inserted on the posterior region, especially close to the posterolateral corner, where a rather dense group is concentrated; at its anterior margin it is as wide as the exposed part of the posterior head capsule, that is, the posterior margin of the DCS; its lateral margin is rounded, strongly widening posteriorly; the posterior edges are rounded; the posterior margin is bisinuate, with a shallow concavity anterad the mesoscutellar shield; the lateral pronotal edge is well pronounced. The pronotal hypomeron is extensive, strongly widening posteriorly; its surface is largely glabrous, with a fine, fingerprint‐like surface pattern; a field of short setae is present in the posterolateral corner, above the femuro‐tibial joint of the foreleg; this area is more concave than the rest of the hypomeron (Figure 6a). The prosternum is as wide as the posterior head region on the ventral side; its surface bears a dense vestiture of adpressed setae, longer than those on most other body regions; the anterior prosternal margin is concave and bears a dense fringe of anteriorly directed fine setae; it is almost parallel‐sided, with the lateral margins only slightly diverging posteriorly; the prosternal region anterior to the procoxae is about half as long as the prosternal width at the anterior margin; the well‐developed prosternal process is moderately broad and posteriorly rounded, and reaches slightly beyond the hind margin of the procoxae; the length of the densely arranged setae is increased on its posterior region; the prosternal part of the procoxal cavity is evenly rounded; the posterolateral edge of the prosternum is oblique and anterolaterally directed; it is adjacent to the well‐developed, distinctly exposed, parallel‐sided protrochantin; the procoxal cavities are posteriorly open (Figure 5a). The proximal part of the trochantinopleura is elongate and flattened, almost band‐shaped; the distal cryptopleural portion is quite large and broad, a slightly curved plate‐like structure which almost reaches the lateral surface of the pronotum and is attached to it by a short muscle (Figure 5a).

##### 3.2.3.1 Endoskeletal Structures

The short profurca originates on the acetabulum of the procoxa with a distinct semiglobular base; this is followed by a moderately narrowed intermediate section; the distal portion is flattened and distinctly widened apically, resembling an axe blade (Figure 5a, b).

##### 3.2.3.2 Fore Legs

Like the other pairs of legs, the fore legs are largely unmodified running legs. They are slightly less than 1 mm long. The procoxa appears subglobular and a considerable portion of it is exposed; like the other leg regions, it bears a regular and dense vestiture of short hairs on most of its exposed surface; a moderately‐sized glabrous projection is present posterolaterally. The well‐developed protrochanter is slightly shorter than the procoxal width and pointed apically. The femur is the largest part of the fore leg; its rounded upper edge fits with the concavity on the posterior pronotal hypomeron; a distinct longitudinal concavity is present on its ventral side for reception of the protibia; it nearly reaches the femoral base and is setose like other surfaces of the leg. The tibia is slightly shorter than the femur and much less voluminous, narrow basally and evenly widening distally; its apicomesal edge bears a distinct straight spur and two smaller curved spurs are present apicolaterally. The five‐segmented tarsus is slightly longer than the tibia; tarsomeres 1–4 are bilobed, equally long, the distal two slightly narrower than the proximal ones; tarsomere 4 is elongate, subcylindrical, and slightly widening distally. Apically a pair of well‐developed, curved, equal claws is present; their base is distinctly widened ventrally and their surface displays indistinct longitudinal riffles; a longitudinal furrow is present on the ventral side (Figure 6d). An empodium with a pair of long setae is present on all legs (in Figure 6d empodial setae are broken off; visible in Figure 6i and especially Figure 6k).

##### 3.2.3.3 Prothoracic Musculature

**FIGURE 7:**
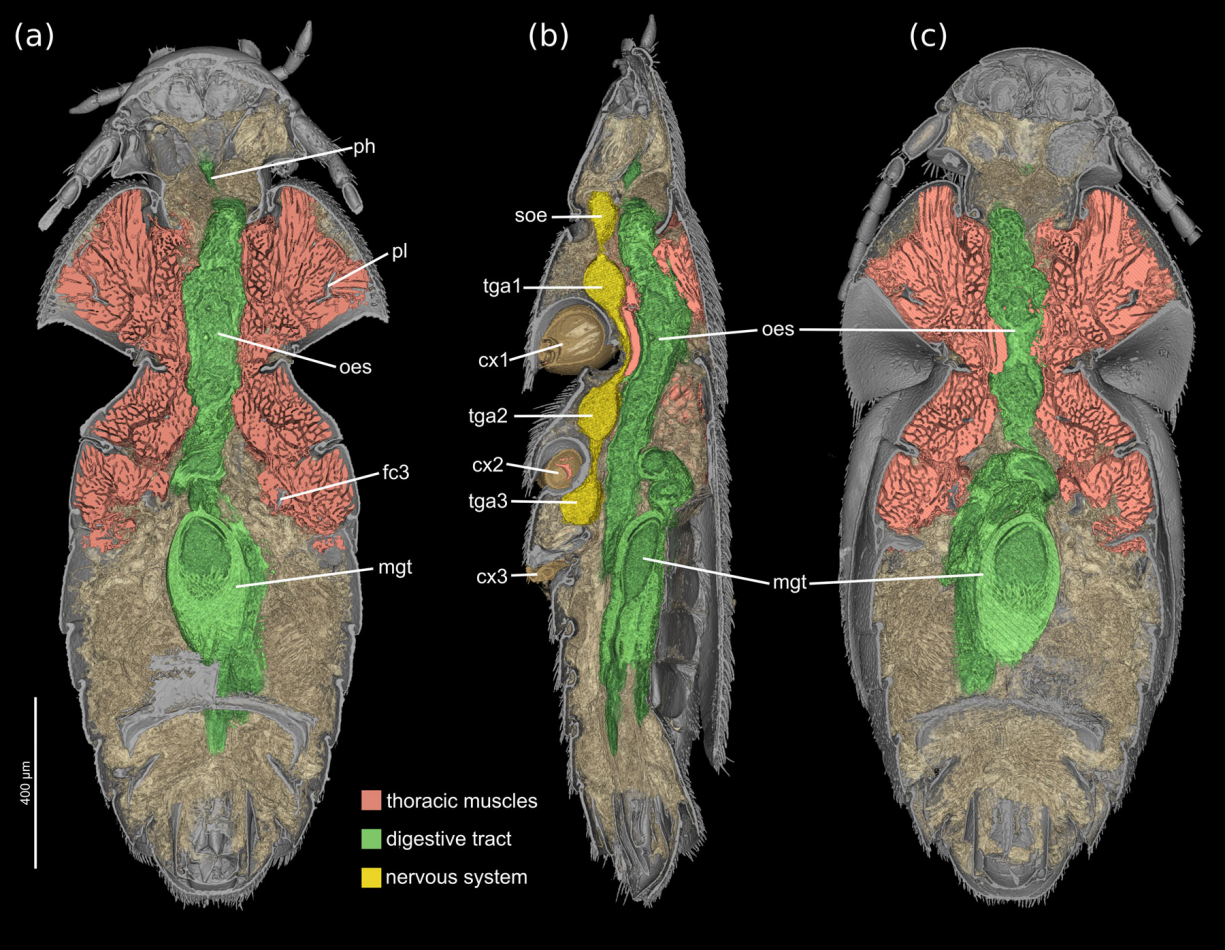
*Silphopsyllus desmanae*, 3D reconstruction of external and internal structures and thoracic musculature, combined with volume renderings of digestive tract, nervous system and other inner organs. (a) dorsal view; (b) lateral view; (c) ventral view. cx1/2/3, pro‐/meso‐/metacoxa; fc3, metafurca; mgt, midgut; oes, esophagus; ph, pharynx; pl, cryptopleura; soe, suboesophageal ganglion; tga1/2/3, pro‐/meso‐/metathoracic ganglion.

**FIGURE 8:**
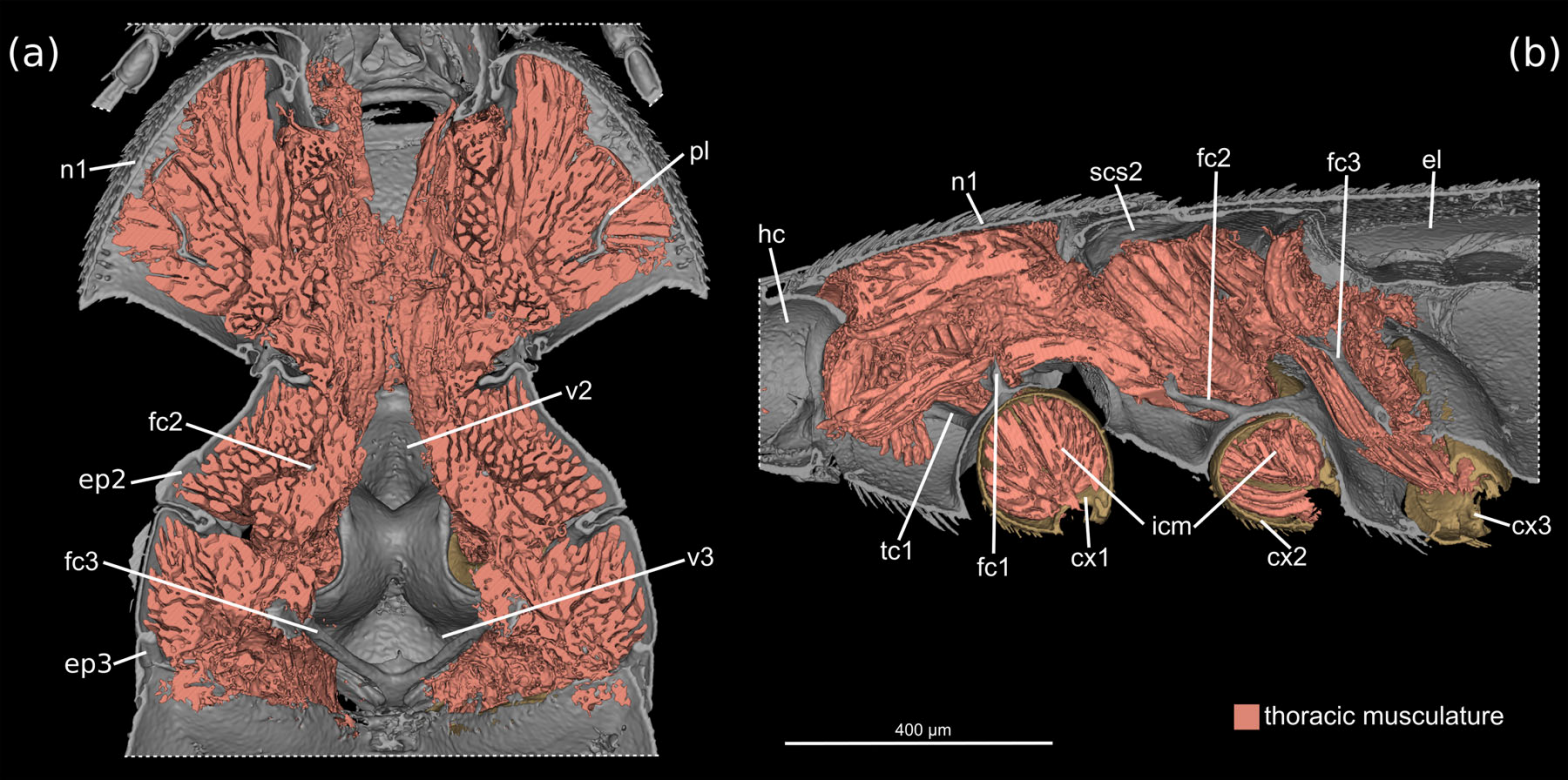
*Silphopsyllus desmanae*, 3D reconstruction of thoracic sclerites and musculature. (a) dorsal view, body horizontally sectioned and (b) sagittal section. cx1/2/3, pro‐/meso‐/metacoxa; el, elytron; ep2/3, mes‐/metepimeron; fc1/2/3, fc1/2/3, pro‐/meso‐/metafurca; hc, head capsule; icm, internal coxal musculature; n1, pronotum; pl, cryptopleura; scs2, mesoscutellar shield; tc1, protrochantin; v2/3, meso‐/metaventrite.

The prothoracic musculature is strongly developed (Figures 7, 8). This includes a full set of neck muscles, for instance muscles originating from the pronotum, the 1st phragma and the profurca, and inserting on the cephalic neck region. The extrinsic and intrinsic leg muscles are also strongly developed, and intersegmental muscles connecting elements of the pro‐ and mesothorax are also present.

#### 3.2.4 Mesothorax

The triangular mesoscutellar shield is exposed and fairly large, about twice as wide as long; it is densely covered with a vestiture of short setae. The mesoventrite anterior to the mesocoxae is slightly shorter than its prothoracic equivalent; its surface is also densely covered with medium length setae. A distinct posteromedian process of the mesoventrite is almost twice as wide as the prosternal process and only slightly shorter; it is nearly parallel‐sided, slightly narrowing towards its apex; it broadly separates the mesocoxae and almost reaches their posterior margin; its apex is truncated, but with rounded posterolateral corners; its surface is densely covered with medium length setae like the prosternum (Figure 6a). The pleural elements are fused with the mesoventrite and with each other, even though faint traces of borders are still recognizable between the ventrite and the mesanepisternum, and the mesanepisternum and mesepimeron, respectively (Figure 6a). The mesocoxal cavities are roughly triangular but with rounded corners.

##### 3.2.4.1 Middle Leg

The middle coxae are similar in shape to the procoxae but slightly larger; the posterolateral process is distinctly larger than its prothoracic counterpart and apically rounded; its surface is smooth and largely glabrous, except for sparsely distributed ventral setae. The distal elements of the leg are similar to those of the proleg in their general configuration (Figure 6e, f). The mesofemur is distinctly larger than the profemur and its posterior margin is weakly concave proximally. The mesotibia is longer than the protibia and bears rows of distinct spines along the lateral margin and the distal half of the mesal margin; longer spines are inserted on the apical tibial region; in addition, two strongly developed spines are present at the apicomesal edge. The tarsus and the five tarsomeres are similar to their prothoracic counterparts but longer; tarsomere 1 is only indistinctly bilobed, narrowed basally and nearly cylindrical distally; tarsomeres 2–4 are also less distinctly bilobed than the equivalent protarsomeres; tenent setae are densely arranged on the ventral surface of the proximal four segments; tarsomere 5 is subcylindrical, slightly widening distally and slightly curved; apically it bears slender, curved, equal claws, slightly longer than those of the prolegs.

##### 3.2.4.2 Elytra

The elytra are well‐developed but do not cover the last two abdominal tergites VII and VIII; posteriorly they are truncated but with evenly rounded posterolateral corners (Figure 1a). The surface is densely covered with setae. A longitudinal carina on the lateral elytral region nearly reaches the apex, while two carinae closer to the mesal margin are only about half as long and less distinct. A broad epipleuron extends to about half of the elytral length. The elytra are widening posteriorly in females. Most of the ventral surface is covered with blunt tubercles ca. 4–5 µm in diameter, but they are almost completely absent on the posterolateral region; the area along the outer posterior and posterior margins are covered with scale‐like microsculpture and sparse adpressed microtrichia.

##### 3.2.4.3 Musculature

The mesothoracic musculature is strongly developed, especially the extrinsic and intrinsic muscles of the middle legs (Figures 7, 8).

#### 3.2.5 Metathorax

The metathorax is unusually small compared to beetles with a well‐developed flight apparatus (e.g., Larsén 1966; Friedrich and Beutel 2010); it is distinctly shorter than the mesothorax and as wide as its posterior region (Figure 5a). The metanotum is strongly simplified; its lateralmost region is bent ventrad and partly overlaps with the metanepisternum (Figure S1a, b); an exposed articulatory area between the lateral scutal margin and the upper metapleuron is thus absent; anteriorly the metanotum slightly overlaps with the mesonotum; all lateral wing processes of the scutum and axillary sclerites are missing; rather shallow, nearly parallel alacristae are preserved, but a distinct scuto‐scutellar border is missing. The metapostnotum is vestigial. The metaventrite is transverse and fused with the pleural elements. The lateral regions are covered with a dense vestiture of fine setae, whereas longer and thicker setae are concentrated in the median area. A short anteromedian process is connected with the corresponding posteromedian process of the mesoventrite; the anterior margin forming the posterior wall of the mesocoxal cavity is obliquely rounded and bears an indistinct bead; the beaded posterior margin is convex laterally; a broad median projection with a median concavity separates the metacoxae (Figure 6a). A longitudinal discrimen and a transverse ridge are not visible, a separate katepisternum thus missing. The metanepisternum and metepimeron are clearly separated by the pleural suture; the former is ventrally fused with the metaventrite, with a faint trace of border still recognizable like in the mesothorax; an upper membranous or semimembranous pleural area is not present and the basalare and subalare are reduced. The metathoracic spiracle is vestigial or absent.

##### 3.2.5.1 Endoskeletal Structures

The well‐developed and elongated mesofurcal arms originate separately on the mesocoxal acetabulum, without a distinctive basal part; the flattened proximal portion is strengthened by a bulge along its mesal edge and extends anteromesad (Figure 5a, b); the distinctly thinner and longer distal portion bends abruptly dorsolaterad and dorsally nearly reaches the upper region of the mesopleuron; the spatulate apical part probably serves as attachment area for a short *M. mesofurca pleuralis*, which stabilizes the entire structure.

##### 3.2.5.2 Hind Legs

The hind legs are distinctly longer than the middle legs (Figure 6g, i). The metacoxae are transverse, inserted in a slightly oblique manner, and distinctly separated medially; a distinct transverse edge is present, approximately parallel to the anterior coxal margin; it separates a short anterior coxal portion from a longer posterior one, but coxal plates are missing; a prominent mesal projection of the coxa is present mesally (Figure 6a); the trochanter articulates with a posterior concavity of this structure, between two rounded processes, a narrower one laterally and a broader one mesally; the lateral metacoxal edge is rounded and does not reach the lateral margin of the ventrite including pleural elements. Most areas of the metacoxae bear a rather sparse vestiture of short and thin setae, but much longer and thicker setae are concentrated on the mesal projection. The distal parts of the leg are similar to the corresponding elements of the middle leg, but larger. The metafemur has a convex posterior margin, like the profemur. The vestiture of the elongate metatibia is similar to that of the mesotibia. The metatarsomeres 1–4 are not bilobed; metatarsomere 5 is elongate and cylindrical; the symmetrical claws at the apex are longer than those of the middle leg (Figure 6k, l).

##### 3.2.5.3 Hind Wings

The hind wings are completely reduced including the axillary sclerites.

##### 3.2.5.4 Endoskeletal Structures

The well‐developed metafurca is longer than the profurca but shorter than the mesofurcal arms; it originates on the short discrimen anterad the metacoxae with a stout common stalk; paired arms extend dorsolaterad (Figure 5a, b); they are distinctly extended apically, forming a concave, pouch‐like muscle attachment area, probably for *M. metafurca‐pleuralis* and *M. metafurca‐intersegmentalis posterior*.

##### 3.2.5.5 Musculature

The metathoracic musculature is distinctly reduced. Notably the large indirect flight muscles, such as dorsal longitudinal muscles or dorsoventral muscles are absent. Due to the suboptimal preservation, we could not unambiguously assess the condition of the small direct flight muscles, but they are likely absent. This is also suggested by the absence of skeletal elements normally associated with the wing base, such as for instance the basalare and its muscle disc and the subalare. The leg muscles are less strongly developed than those of the foreleg and middle leg.

#### 3.2.6 Abdomen

The abdomen displays six distinctly visible segments in ventral view (Figures 1, 5). The shortened elytra leave the posterodorsal abdomen exposed, the entire terminal tergite VIII, a large portion of tergite VII, laterotergite VII, a large portion of laterotergite VI, and a small part of laterotergite V. Tergite VIII is roughly trapezoidal in shape but with rounded posterolateral edges; its surface is densely covered with short setae like the other exposed parts of the dorsal side of the abdomen. Tergite VII is distinctly larger than VIII, parallel‐sided and distinctly concave at its posterior margin. Tergite I is weakly sclerotized; laterally it partly covers the large and annular spiracle I. The following tergites II‐VI are smooth and glabrous, about equal in length, transverse, with parallel anterior and posterior margins. Distinct laterotergites are present on the dorsal side of segments II‐VII; they are triangular and posteriorly pointed on segment VII, but otherwise quadrangular; all of them bear a dense vestiture of short setae. The first exposed ventrite, that is, sternite III, and the four following ones bear a dense vestiture of medium length and long setae, with the latter more concentrated on the posterior ventrites except for the terminal one. The terminal ventrite VI is of parabolic shape in females; it is glabrous medially but setose laterally; posteromedially it displays a deep incision with a median triangular projection.

##### 3.2.6.1 Musculature

As in the other tagmata the state of preservation did not allow for a detailed reconstruction of the abdominal musculature. However, the µCT data set suggests that the arrangement of the muscles is similar to the condition observed in *Platypsyllus* (Yavorskaya et al. 2023).

##### 3.2.6.2 Ganglionic Chain

The postcephalic ganglionic chain is well developed (Figure 7b). The fairly small terminal ganglionic complex VIII is located in the region of abdominal segment IV. A distinct pair of nerves extends into the posterior abdomen.

### 3.3 Results of the Cladistic Analysis

**FIGURE 9:**
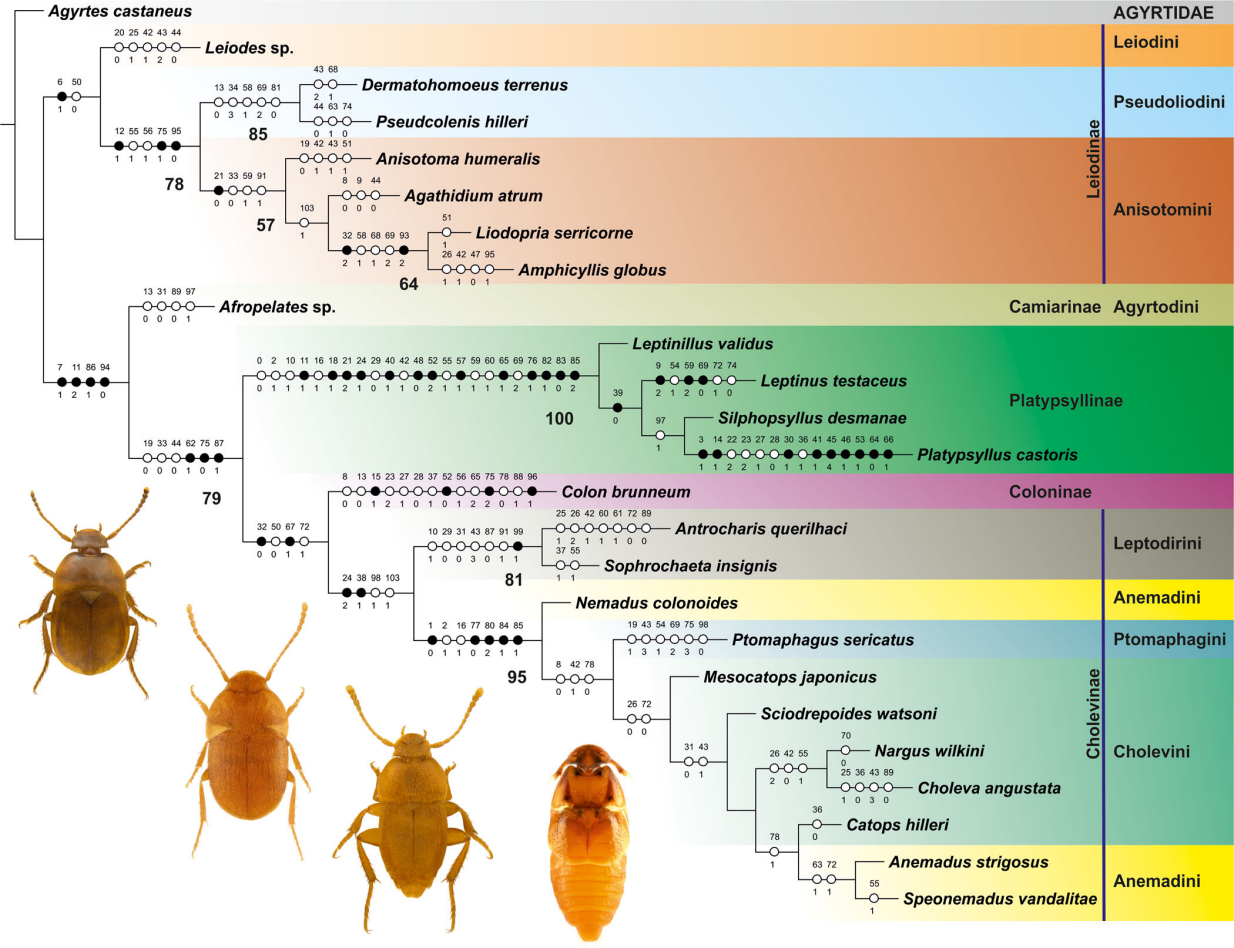
Phylogenetic relationships in Leiodidae. Cladistic analysis using TNT, tree 2 of two minimum length trees, with character state mapping and bootstrap values; length L = 257, retention index Ri = 0.76, and consistency index Ci = 0.52. Full circles indicate non‐homoplasious changes, and empty circles homoplasious transformations; bootstrap values below 50 not shown. Dorsal habitus photographs show (from left to right) *Leptinillus validus*, *Leptinus testaceus*, *Silphopsyllus desmanae*, and *Platypsyllus castoris*.

The analysis yielded two most parsimonious trees after using implied weighting, differing only in the topology within Platypsyllinae: tree 1 shows a pattern *Leptinillus* + (*Silphopsyllus* + (*Leptinus* + *Platypsyllus*)), tree 2 *Leptinillus* + (*Leptinus* + (*Silphopsyllus* + *Platypsyllus*)). Tree 2 was selected for the character mapping and discussion (Figure 9), because the sister group relationship between *Leptinus* and *Platypsyllus*, based on morphological features, seems less likely than that of *Silphopsyllus* and *Platypsyllus* (based on the broad host spectrum, *Leptinus* could even be a better candidate for the most “basal genus” in the subfamily than *Leptinillus*; see Discussion). The length of the obtained trees is L = 257, the retention index Ri = 0.76, and the consistency index Ci = 0.52. The camiarine genus *Afropelates* was resolved as sister to Platypsyllinae + (Coloninae + Cholevinae), and this topology is supported by four apomorphies (in brackets character and character state): distance between eye (or ocular prominence if eyes are lacking but their sites marked by an elevated area) and antennal fossa subequal to distance between eye and posterior margin of temple [7(1)]; supraantennal ridges forming circular or semicircular edges of antennal fossae [11(2)]; mesocoxa small, at most as long as half width of mesofemur [86(1)]; and protarsi in females distinctly flattened and broadened [94(0)]. Platypsyllinae were resolved as sister to Coloninae + Cholevinae, and this relationship is supported by multiple setae on the mesoscutellar shield [62(1)]; the anterior metaventral process absent [75(0)]; and the metacoxa in ventral view subrectangular [87(1)]. The Platypsyllinae are monophyletic and supported by a large set of 13 non‐homoplasious apomorphies: supraantennal ridges directed mesad but not extending beyond antennal insertions and not forming an uninterrupted transverse ridge [11(1)]; medially continuous laminatentorium absent [18(1)]; submentum subrectangular and delimited by lateral 'sutures‘ [21(2)]; labrum 1.5–2 times as broad as mentum and subequal in width to maxillary‐labial complex [24(1)]; posteriorly projecting lateral lobes of mentum present [40(1)]; periarticular gutters on antennomeres absent [48(0)]; cervical sclerites absent [52(2)]; precoxal region of prosternum much longer than coxal rests [57(1)]; placement of mesocoxal cavities closer to midline than to lateral margins of mesoventrite [65(1)]; metanepisterna fused with metaventrite [76(1)]; metascutum laterally overlapping meso‐ and metapleural regions [82(1)]; exposed portion of procoxa subglobose or only slightly elongate [83(0)]; and mesocoxa subglobose [85(2)]. Within Platypsyllinae, *Leptinus* + (*Silphopsyllus* + *Platypsyllus*) share the subquadrate or subrectangular mentum [39(0)] and completely reduced eyes (not included in the analysis). *Platypsyllus* is a highly derived genus, showing many character states not found in any other analyzed taxa: specialized ctenidial setae along posterior margin of vertex present [3(1)]; cephalic cavity to receive maxillary palp present [14(1)]; long subapical or submedian seta on maxillary palpifer absent [30(1)]; anterior margin of mentum distinctly but evenly and not conspicuously deeply concave [41(1)]; antennal insertion posterolateral, exposed in posterolateral view [45(4)]; antenna composed of nine antennomeres [46(1)]; dorsolateral groove on pronotum to receive antenna present [53(1)]; elytral epipleurae inconspicuous and narrow, almost absent [64(0)]; and protrochantin concealed [66(1)].

## 4 Discussion

### 4.1 Phylogenetic Position of *Silphopsyllus*

The placement of *Silphopsyllus desmanae* in monophyletic Platypsyllinae is strongly supported by numerous apomorphic features of this specialized subunit of Leiodidae (see 3.3. Results of the cladistic analysis). The most conspicuous of them is arguably the presence of long lobes of the mentum projecting posteriorly. In addition, the firm connection between the clypeus and labrum, the latter lacking tormae and the usual connecting membrane, a distinct level of depigmentation of the cuticle, a distinctly flattened body, the posteromedian cephalic projection (PMCP), and an unusually small maxilla (not included in the phylogenetic analysis here) are also potential autapomorphies of Platypsyllinae (see discussion in Yavorskaya et al. (2023)). The partial (*Leptinillus*) reduction of the compound eyes is likely an additional apomorphic groundplan feature of the subfamily. In contrast to Yavorskaya et al. (2023), the dorsal cephalic shield (DCS) and posterior cephalic projection (PCP) are not autapomorphies of Platypsyllinae, but have already evolved earlier within Leiodidae. They are distinctly developed in *Catops* and other members of the family (Antunes‐Carvalho et al. 2017a). Another unique apomorphy of Platypsyllinae is an unusually robust pro‐mesothoracic connecting membrane, making it extremely difficult to disarticulate the head + prothorax from the pterothorax. This feature was already noticed by Hlavac (1972) and discussed for *Platypsyllus* by Yavorskaya et al. (2023). Here we confirm that all genera of Platypsyllinae share this important functional adaptation that increases the body integrity.

Within Platypsyllinae, *Platypsyllus* has evolved numerous derived features missing in the three other genera (Yavorskaya et al. 2023), for instance specialized ctenidial setae along the posterior margin of the vertex, the antennal insertion shifted posterolaterad and exposed in posterolateral view, the unusually shaped antenna composed of only nine antennomeres, the dorsolateral antennal groove on the pronotum, the concealed protrochantin, vertical cuticular columns in the head and various thoracic intrasegmental strengthening ridges, the loss of the secondary mandibular joint, the strongly shortened elytra, laterally widening metacoxae, a broadened metafemur, enlarged tibial spurs, specialized rows of hairs on the abdominal sternites, and a retracted abdominal tergite VII. A strongly condensed abdominal ganglionic chain with all ganglia except those of segments I and II fused is likely another autapomorphy of the genus, but anatomical data for *Leptinillus* and *Leptinus* are not available yet.

The placement of Platypsyllinae within Leiodidae, and the internal phylogenetic topology of this family are not firmly established yet. Fresneda at al. (2011) resolved Platypsyllinae + Leiodinae as a sister group to Cholevinae, but their analysis was focused on relationships within Leptodirini (Cholevinae), and did not include other subfamilies of Leiodidae. McKenna et al. (2015) placed *Colon* outside Leiodidae, as the sister group to Hydraenidae + Ptiliidae, and *Platypsyllus* as sister to a clade formed by genera currently included in Anisotomini, Pseudoliodini and Scotocryptini (Leiodinae). Antunes‐Carvalho et al. (2017b), in a parsimony analysis with implied weights, resolved *Leptinus* within Cholevinae, with Oritocatopini + Eucatopini sister to *Leptinus* + all remaining cholevines, while in a Bayesian analysis *Leptinus* + *Catopocerus* [Catopocerinae] were placed as sister to *Eupelates* [Agyrtodini] + (*Agyrtodes* [Agyrtodini] + Cholevinae). These are only examples of distinctly different results obtained in recent phylogenetic reconstructions based on morphological (Antunes‐Carvalho et al. 2017b) or molecular data sets (McKenna et al. 2015), or analyses based on either of them (Fresneda et al. 2011). In our analysis, the placement of Platypsyllinae, based on morphological characters of all included genera, as a sister group to Coloninae + Cholevinae is supported by multiple setae present on the mesoscutellar shield, the anterior metaventral process highly reduced, and a subrectangular metacoxa. These are relatively weak apomorphies and a larger taxon sampling, including all tribes of Leiodidae is needed to verify this result. As the anatomical data for the subfamily are still fragmentary, the phylogenetic interpretation we present here is preliminary. With the evidence at hand, a placement of *Leptinillus* as sister to the remaining Platypsyllinae is the most parsimonious solution, and a sister group relationship between *Silphopsyllus* and *Platypsyllus* seems more probable than between *Leptinus* and *Platypsyllus*. It is clear that *Platypsyllus* is the genus with the maximum of apomorphic features, showing a number of far‐reaching morphological modifications related to its mode of life (discussed in Yavorskaya et al. 2023). Alternatively, the comparatively broad host spectrum of *Leptinus* tentatively suggests that it may be the most “basal genus” in the subfamily. However, this is not supported by the phylogenetic analysis of our morphological data. Moreover, as different hosts are used by species of the other three genera, host specialization cannot be a synapomorphy of Platypsyllinae excl. *Leptinus*. Future analyses of suitable molecular data may reveal the true relationships in the group with three morphologically similar genera.

### 4.2 Adaptations of Platypsyllinae to Mammalian Hosts

Three species of Platypsyllinae are specialized on a single mammalian host, two of them more or less closely associated with water (e.g., Peck 2007). *Silphopsyllus desmanae* is only known from the semiaquatic Russian desman (Olsufiev 1923; Pavlovsky 1956), and *Platypsyllus castoris* and *Leptinillus validus* from the beaver, the former with a Holarctic distribution, the latter restricted to North America (Peck 2007). *Leptinillus aplodontiae* occurs only on *Aplodontia rufa* (Aplodontiidae), a small mammal known as the “mountain beaver,” earlier considered as close to the root of rodents (e.g., Peck 2007: “ancestral status in Rodentia”) but in fact likely closely related with squirrels (Piaggio et al. 2013). In contrast to these species, *Leptinus testaceus* has a broad spectrum of hosts, comprising species of the rodent family Muridae and of the non‐related Soricidae (or shrews) of the order Eulipotyphla. The occasional occurrence in bumble bee nests is likely a coincidence according to Peck (2007).

**FIGURE 10:**
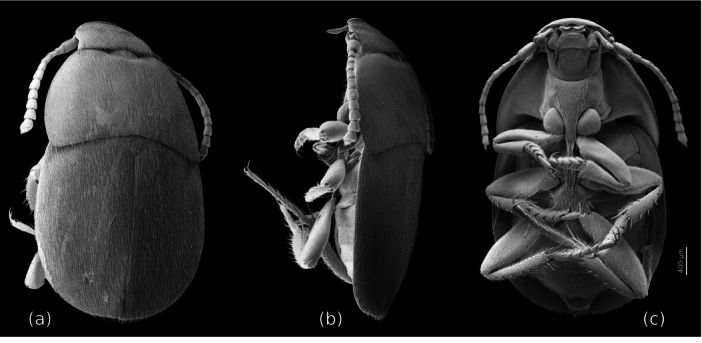
*Leptinillus validus*, scanning electron micrographs, habitus. (a) dorsal view; (b) lateral view; (c) ventral view.

Even though the host of *Silphopsyllus desmanae* is closely associated with water, arguably only slightly less so than beavers in the case of the closely related *Platypsyllus castoris* (Winter 1979), the morphological adaptations are distinctly less advanced in the Russian species. The head and postcephalic body of *Silphopsyllus* are less distinctly flattened than in *Platypsyllus* and the stabilization and immobilization of the head capsule less advanced. A highly unusual complex of apomorphies of *Platypsyllus* listed in the previous chapter is lacking in *Silphopsyllus* and the other two genera of Platypsyllinae (Jeannel 1922; Wood 1965; Yavorskaya et al. 2023), a far‐reaching reinforcement of the skeleton, with a greatly increased thickness of the cuticle and a unique set of vertical cuticular columns in the head (ADVC, PDVC) (Yavorskaya et al. 2023: Figure 6). Apparently, beavers are capable of much more forceful grooming than the distinctly smaller vykhukhol’, or the mechanical strain imposed on the beetle's body moving forward in the extremely dense beaver's fur is greater than that on the commensal of the desman. Anchorage and moving in the fur of the host is clearly optimized in *Platypsyllus* compared to *Silphopsyllus*, with various morphological adaptations described in detail in Yavorskaya et al. (2023) (see also Winter 1979). Considering this, it may appear surprising that the north American species *Leptinillus validus*, one of the two species of Platypsyllinae with the least specialized morphology, can cope with the beaver as a host just like *Platypsyllus* (Peck 2007) (Figure 10). However, it was emphasized by Peck (2007) that this species spends more time in the host's lodge and off the host's body than on it. This suggests that a loose association with small or medium sized mammals belongs to the groundplan of Platypsyllinae, and that a closer attachment to a single water‐associated host has evolved in the genera *Platypsyllus* and *Silphopsyllus*, which are possibly sister taxa.

## 5 Conclusions

The morphological part of our study confirms that *Silphopsyllus* shares a large number of derived features with other platypsyllines, but is much less specialized morphologically than the closely related *Platypsyllus*. The monophyly of the subfamily is confirmed. Our analysis suggests a phylogenetic arrangement *Leptinillus* (+ *Leptinus* + (*Silphopsyllus* + *Platypsyllus*)), but a closer relationship between *Leptinus* and *Platypsyllus* cannot be excluded. Platypsyllinae is placed as sister to Coloninae + Cholevinae within Leiodidae, based on several putative morphological synapomorphies. We suggest that ancestral platypsyllines were loosely associated with small or medium‐sized mammals, whereas a close association with different semiaquatic mammalian hosts evolved in *Platypsyllus* and *Silphopsyllus*, either independently or less likely as a shared derived groundplan feature, that is, synapomorphy.

## Author Contributions

**Paweł Jałoszyński:** conceptualization, methodology, investigation, writing–original draft, writing–review and editing, formal analysis, software. **Odair M Meira:** investigation, visualization, software. **Margarita I Yavorskaya:** conceptualization, data curation, methodology, investigation, visualization, writing–review and editing, software. **Alexandr Prokin:** writing–review and editing, resources. **Veit Grabe:** visualization, investigation. **Rolf G Beutel:** conceptualization, project administration, investigation, writing–original draft, writing–review and editing.

### Peer Review

The peer review history for this article is available at https://www.webofscience.com/api/gateway/wos/peer-review/10.1002/jmor.70031.

## Supporting information

S1: *Silphopsyllus* SEM micrographs, elytra.

S2: *Leptinillus* SEM micrographs, head structures.

S3: List of characters.

S4: Character state matrix.

## Data Availability

The data that supports the findings of this study are available in the supplementary material of this article. The micro‐CT data set investigated in this study will be uploaded in a Zenodo repository.

## References

[jmor70031-bib-0001] Antunes‐Carvalho, C. , I. Ribera , R. G. Beutel , and P. Gnaspini . 2017b. “Morphology‐Based Phylogenetic Reconstruction of Cholevinae (Coleoptera: Leiodidae): A New View on Higher‐Level Relationships.” Cladistics 35: 1–41. 10.1111/cla.12230. 34636438

[jmor70031-bib-0002] Antunes‐Carvalho, C. , M. Yavorskaya , P. Gnaspini , I. Ribera , J. U. Hammel , and R. G. Beutel . 2017a. “Cephalic Anatomy and Three‐Dimensional Reconstruction of the Head of *Catops ventricosus* (Weise, 1877) (Coleoptera: Leiodidae: Cholevinae).” Organisms Diversity and Evolution 17: 199–212. 10.1007/s13127-016-0305-3.

[jmor70031-bib-0003] Bánki, O. , Y. Roskov , and M. Döring . 2024. Catalogue of Life (Version 2024‐08‐29). Catalogue of Life, Amsterdam, Netherlands. 10.48580/dgdwl.

[jmor70031-bib-0004] Barabash‐Nikiforov, I. I. 1950. Beaver and Desman as Components of Water‐Shore Complex, 107. Voronezh: Voronezh State University Publishers.

[jmor70031-bib-0005] Beutel, R. G. , S. G. Friedrich , S.‐Q. Ge , and X.‐K. Yang . 2014. Insect Morphology and Phylogeny, 516. Berlin, Boston: Walter de Gruyter.

[jmor70031-bib-0006] Engelkes, K. , F. Friedrich , J. U. Hammel , and A. Haas . 2018. “A Simple Setup for Episcopic Microtomy and a Digital Image Processing Workflow to Acquire High‐Quality Volume Data and 3D Surface Models of Small Vertebrates.” Zoomorphology 137: 213–228. 10.1007/s00435-017-0386-3.

[jmor70031-bib-0007] Fresneda, J. , V. Grebennikov , and I. Ribera . 2011. “The Phylogenetic and Geographic Limits of Leptodirini (Insecta: Coleoptera: Leiodidae: Cholevinae), With a Description of *Sciaphyes shestakovi* sp.n. From the Russian Far East.” Arthropod Systematics & Phylogeny 69: 99–123.

[jmor70031-bib-0008] Friedrich, F. , and R. G. Beutel . 2010. “Goodbye Halteria? The Thoracic Morphology of Endopterygota (Insecta) and Its Phylogenetic Implications.” Cladistics 26: 579–612. 34879598 10.1111/j.1096-0031.2010.00305.x

[jmor70031-bib-0009] Goloboff, P. A. , J. S. Farris , and K. C. Nixon . 2008. “TNT, a Free Program for Phylogenetic Analysis.” Cladistics 24: 774–786.

[jmor70031-bib-0010] Hlavac, T. F. 1972. “The Prothorax of Coleoptera: Origin, Major Features of Variation.” Psyche: A Journal of Entomology 79: 123–149.

[jmor70031-bib-0011] Jeannel, R. 1922. “Morphologie comparée du *Leptinus testaceus* Müll. et du *Platypsyllus castoris* Rits. *Biospeologica XLV* .” Archives de Zoologie Expérimentale et Générale 60: 557–592.

[jmor70031-bib-0025] Kéler, S. v. 1963. Entomologisches Wörterbuch. Berlin: Akademie Verlag.

[jmor70031-bib-0012] Kennerley, R. , and S. T. Turvey 2016. *Desmana moschata*. *The IUCN Red List of Threatened Species* 2016: e.T6506A22321477. 10.2305/IUCN.UK.2016-2.RLTS.T6506A22321477.en.

[jmor70031-bib-0013] Larsén, O. 1966. “On the Morphology and Function of Locomotor Organs of the Gyrinidae and Other Coleoptera.” Opuscula Entomologica, Supplementum 30: 1–241.

[jmor70031-bib-0014] Lösel, P. D. , T. van de Kamp , A. Jayme , et al. 2020. “Introducing Biomedisa as an Open‐Source Online Platform for Biomedical Image Segmentation.” Nature Communications 11: 5577. 10.1038/s41467-020-19303-w PMC7642381 33149150

[jmor70031-bib-0015] McKenna, D. D. , B. D. Farrell , M. S. Caterino , et al. 2015. “Phylogeny and Evolution of Staphyliniformia and Scarabaeiformia: Forest Litter as a Stepping Stone for Diversification of Nonphytophagous Beetles.” Systematic Entomology 40: 35–60. 10.1111/syen.12093.

[jmor70031-bib-0016] Nixon, K. C. 1999‐2002. WinClada, v. 1.00.08. Software published by the author, Ithaca, NY, USA. [online]. https://www.scienceopen.com/document?vid=3c4c21f5-f2fc-49b1-a18e-4fba2f1337d7. [accessed in November 2020].

[jmor70031-bib-0017] Olsufiev, G. 1923. “ *Silphopsyllus desmanae*, gen. et sp. n. (Coleoptera, Leptinidae), parasit vyckuckoli. *Silphopsyllus desmanae*, gen. et sp. n. (Coleoptera, Leptinidae), parasite du rat musqué.” Entomologicheskoe Obozrenie 18: 81–90.

[jmor70031-bib-0018] Page, R. D. M. 2001. NDE: NEXUS Data Editor 0.5.0. University of Glasgow. http://taxonomy.zoology.gla.ac.uk/rod/NDE/nde.html [accessed in December 2010].

[jmor70031-bib-0019] Pavlovsky, E. N. 1956. “On the Anatomy of *Silphopsyllus desmanae* Ols. (Coleoptera, Leptinidae.” Revue d'entomologie de l'USSR 30: 518–529.

[jmor70031-bib-0020] Peck, S. B. 2007. “Distribution and Biology of the Ectoparasitic Beetles *Leptinillus validus* (Horn) and *L. aplodontiae* Ferris of North America (Coleoptera: Leiodidae: Platypsyllinae).” Insecta Mundi 3: 1–7.

[jmor70031-bib-0021] Piaggio, A. J. , B. A. Coghlan , A. E. Miscampbell , W. M. Arjo , D. B. Ransome , and C. E. Ritland . 2013. “Molecular Phylogeny of an Ancient Rodent Family (Aplodontiidae).” Journal of Mammalogy 94: 529–543.

[jmor70031-bib-0022] Prokin, A. A. , and A. I. Zemlyanukhin . 2008. “About Finding of *Silphopsyllus desmanae* Olsufiev, 1923 (Coleoptera, Leiodidae, Platypsyllinae) in the Lipetsk Oblast’. In: Condition and Problems of the Middle Russian Forest‐Steppe Ecosystems (Voronezh).” Proceedings of the Venevitinovo Research‐Educational Center of Voronezh State University 21: 121–123.

[jmor70031-bib-0023] Schneeberg, K. , R. Bauernfeind , and H. Pohl . 2017. “Comparison of Cleaning Methods for Delicate Insect Specimens for Scanning Electron Microscopy.” Microscopy Research and Technique 80: 1199–1204. 28802096 10.1002/jemt.22917

[jmor70031-bib-0024] Semenov‐Tian‐Shansky, A. , and Th. Dobzhansky . 1927. “Die Larve von *Silphopsyllus desmanae* Ols., Parasit der Moschusratte, als Kriterium seiner genetischen Beziehungen und seiner systematischen Stellung.” Revue Russe d'Entomologie 21: 8–16.

[jmor70031-bib-0026] Winter, G. 1979. “Untersuchungen zur Morphologie des Biberkäfers *Platypsyllus castoris* Ritsema 1869 (Coleoptera), eines extrem gut angepassten Vertreters des Lebensformtyps fellbewohnender Insekten.” Zoologische Jahrbücher. Abteilung für Anatomie und Ontogenie der Tiere 101: 456–471.

[jmor70031-bib-0027] Wood, D. M. 1965. “Studies on the Beetles *Leptinillus validus* (Horn) and *Platypsyllus castoris* Ritsema (Coleoptera: Leptinidae) From Beaver.” Proceedings of the Entomological Society of Ontario 95: 33–63.

[jmor70031-bib-0028] Yavorskaya, M. I. , P. Jałoszyński , and R. G. Beutel . 2023. “A Unique Case of commensalism: The Beaver Beetle *Platypsyllus castoris* (Leiodidae, Coleoptera) and Its Morphological Adaptations.” Journal of Morphology 284: e21532. 36317298 10.1002/jmor.21532

[jmor70031-bib-0029] Zemlyanukhin, A. I. 2009. Russian Desman in the Lipetsk Oblast, 104. Lipetsk: Lipetsk State Pedagogical University Publishers.

