# Supplementary figures and images for: The Morphology of the Rare Beetle Silphopsyllus desmanae (Leiodidae), a Commensal of the Semiaquatic Russian Desman
Source: J Morphol. 2025 Feb 26;286(3):e70031. doi: 10.1002/jmor.70031 (PMC11865005; doi:10.1002/jmor.70031)

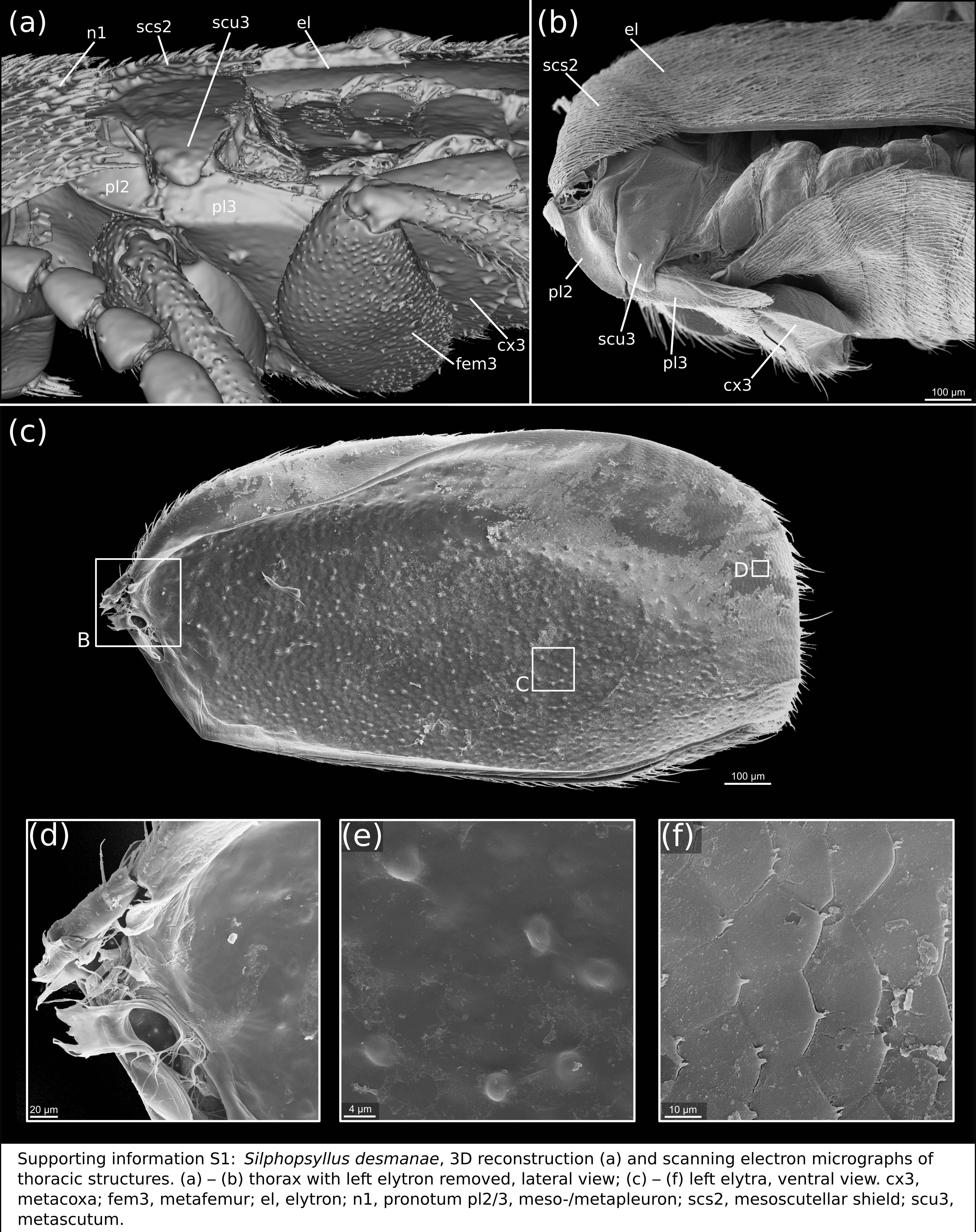

Supplement: Supplementary file 1 — S1: Silphopsyllus SEM micrographs, elytra. [file JMOR-286-e70031-s004.png]

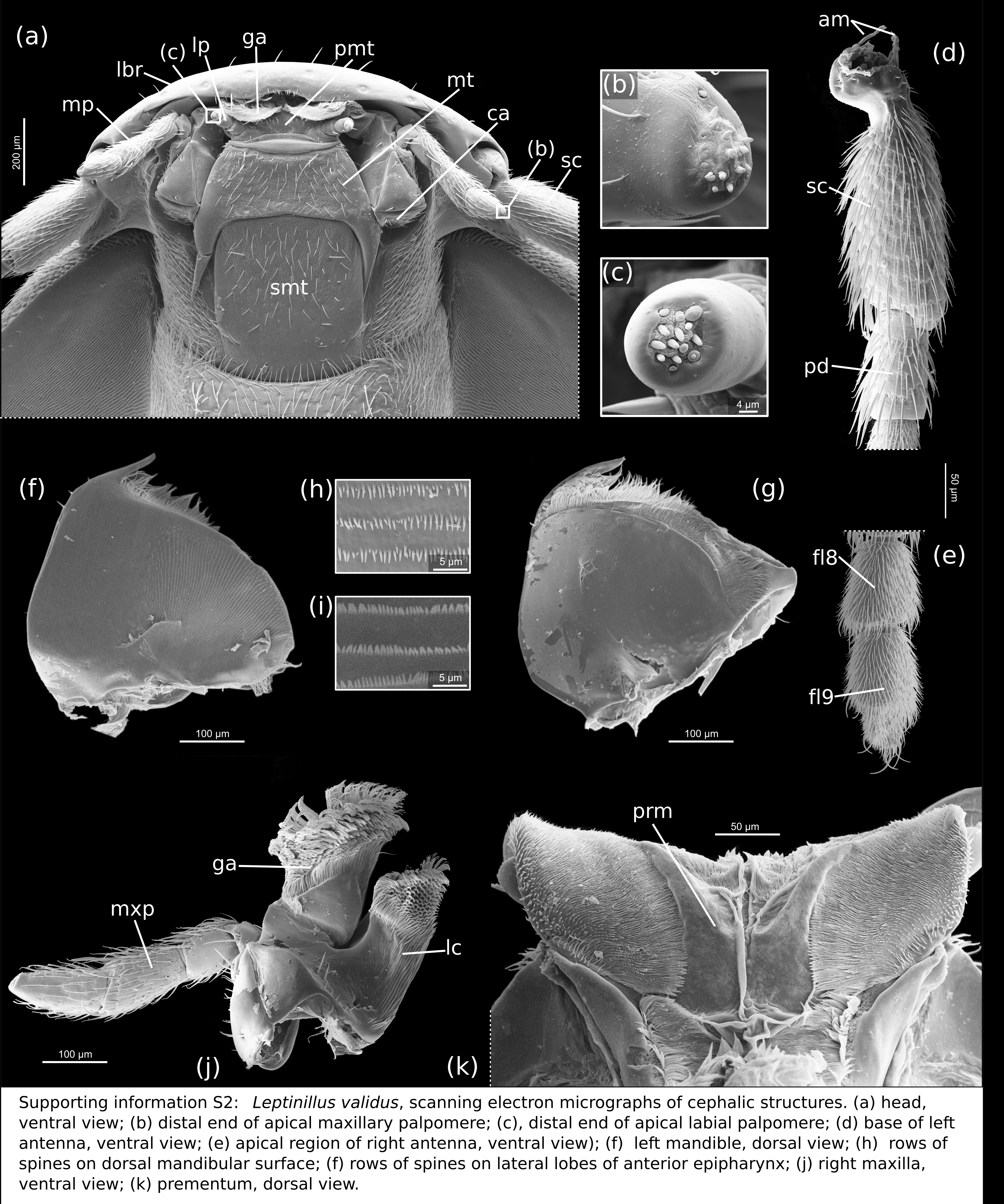

Supplement: Supplementary file 2 — S2: Leptinillus SEM micrographs, head structures. [file JMOR-286-e70031-s002.png]
