# Supplementary material for: The Morphology of the Rare Beetle Silphopsyllus desmanae (Leiodidae), a Commensal of the Semiaquatic Russian Desman
Source: J Morphol. 2025 Feb 26;286(3):e70031. doi: 10.1002/jmor.70031 (PMC11865005; doi:10.1002/jmor.70031)
Supplement: Supplementary file 3 — S3: List of characters. [file JMOR-286-e70031-s001.rtf]

Characters and character states used in the cladistic analysis:

0.	Structure of head capsule: (0) anterior region and neck nearly in one plane (Fig. 2c); (1) with oblique but distinct angle (concave ventrally) between anterior region and neck (e.g. Antunes-Carvalho et al. 2016: Fig. 6a).
1.	Head capsule in lateral view: (0) approximately hourglass-shaped, clearly subdivided into posterior and anterior regions by dorsal and ventral impressions (e.g. Antunes-Carvalho et al. 2017a: Fig. 6a); (1) wedge-shaped, approximately gradually broadening posteriorly (Fig. 2c).
2.	Vertex and occiput: (0) confluent (e.g. Newton 1997: Fig. 2); (1) demarcated by sharply marked posteriorly-projecting transverse ridge (vertexal crest) that tightly adheres to anterior pronotal margin (e.g. Antunes-Carvalho et al. 2017a: Fig. 2a).
3.	Ctenidial setae along posterior margin of vertex: (0) absent (e.g. Antunes-Carvalho et al. 2017a: Fig. 2a); (1) present (Yavorskaya et al. 2022: Fig. 2a).
4.	Supraocular groove along upper mesal margin of eye: (0) absent; (1) present (indicated as “supraocular ridge” above the groove in Wheeler & Miller 2005: Fig. 10).
5.	In lateral view, middle of eye: (0) at or above middle of head height (e.g. Wheeler & Miller 2005: Fig. 10); (1) below middle of head height (e.g. Antunes-Carvalho et al. 2017a: Fig. 2e).
6.	Distance between eye (or ocular prominence if eyes are lacking but their sites marked by an elevated area) and mandibular base: (0) longer than distance between eye and posterior margin of temple (e.g. Antunes-Carvalho et al. 2017a: Fig. 2e); (1) shorter than distance between eye and posterior margin of temple (e.g. Wheeler & Miller 2005: Fig. 10); (2) subequal to distance between eye and posterior margin of temple.
7.	Distance between eye (or ocular prominence if eyes are lacking but their sites marked by an elevated area) and antennal fossa: (0) longer than distance between eye and posterior margin of temple (e.g. Antunes-Carvalho et al. 2017a: Fig. 2e); (1) subequal to distance between eye and posterior margin of temple; (2) shorter than distance between eye and posterior margin of temple (e.g. Wheeler & Miller 2005: Fig. 10).
8.	Frontoclypeal border: (0) not marked (e.g. Antunes-Carvalho et al. 2017a: Fig. 2a); (1) marked by distinct inflection (e.g. Zhou et al. 2005: Fig. 2a); (2) not marked on surface or marked as a fine ridge, in both cases internal ridge visible through cuticle (Fig. 2c, sagittal section through transverse ridge visible between clypeus and frontal region).
9.	Width of clypeus (scored in females, if in males clypeus modified): (0) 3-6 times as wide as long; (1) ca. 8 times as wide as long; (2) 10 times and more as wide as long; (3) 2-2.5 times as broad as long; (4) 1-1.5 times as broad as long.
10.	Antennal insertions: (0) close to anterior tentorial pits and to internal frontoclypeal ridge; (1) shifted strongly posteriorly and distant from anterior tentorial pits and interior frontoclypeal ridge (e.g. Æurèiæ et al. 2023: Fig. 7c).
11.	Supraantennal ridges: (0) directed anteromesally and anteriorly broadly separated (e.g. Newton 1997: Fig. 2); (1) directed mesally but not extending beyond antennal insertions and not forming one transverse ridge (Fig. 3a); (2) forming circular or semicircular edges of antennal fossae (e.g. Antunes-Carvalho et al. 2017a: Fig. 2e).
12.	Antennal sulcus or at least distinct, smooth impression along anterior (and often extending toward lateroventral) margin of eye: (0) absent; (1) present (e.g. “antennal groove” of Wheeler & Miller 2005: Fig. 10).
13.	Border between tempora and occiput: (0) not marked or marked only by different surface sculpture and/or setation; (1) marked by abrupt or gradual narrowing (e.g. Antunes-Carvalho et al. 2017a: Fig. 2a).
14.	Cephalic cavity to receive maxillary palp: (0) absent; (1) present, ventrolateral (Yavorskaya et al. 2022: Fig. 2c).
15.	Neck region of head: (0) clearly defined but short, not forming large ball-and-socket joint with prothorax (e.g. Antunes-Carvalho et al. 2017a: Fig. 2a); (1) not differentiated, head gradually narrowing from posterior margins of eyes (but a short occipital collar may be present).
16.	Width of neck region of head: (0) subequal in width to anterior region of head excluding eyes (e.g. Wheeler & Miller 2005: Fig. 12); (1) distinctly narrower than anterior region of head (e.g. Antunes-Carvalho et al. 2017a: Fig. 2a).
17.	Neck region narrower than anterior portion of head: (0) distinctly broader than half width of anterior portion of head; (1) as broad as 1/3-1/4 width of anterior region of head.
18.	Lateral components of tentorium anterior to tentorial bridge: (0) with laminatentoria connected at middle (Fig. 2d); (1) not connected (Fig. 2e).
19.	Anterolateral regions of submentum: (0) not projecting anteriorly or only weakly convex anterolaterally (e.g. Antunes-Carvalho et al. 2017a: Fig. 2c); (1) projecting anteriorly, anterolaterally or laterally forming anterolateral lobes (e.g. Wheeler & Miller 2005: Fig. 11, but incorrectly annotated as gular sutures).
20.	Lateral submental 'sutures': (0) absent; (1) present (e.g. Wheeler & Miller 2005: Fig. 11, but submentum annotated as gula).
21.	Submentum delimited by lateral 'sutures': (0) subtriangular; (1) subtrapezoidal; (2) subrectangular.
22.	Gular sutures: (0) narrowly separated; (1) broadly separated (Fig. 1c); (2) obliterated at least at and partly behind posterior tentorial pits / area of insertion of posterior tentorial arms.
23.	Largest width of gular plate at posterior margin of head: (0) subequal to 1/3 width of occiput / neck region; (1) at least as wide as half width of occiput / neck region; (2) gular plate indiscernible or not demarcated laterally.
24.	Width of labrum: (0) subequal to width of mentum, clearly much narrower than width of entire maxillary-labral complex; (1) 1.5-2 times as broad as mentum and subequal to width of maxillary-labial complex; (2) distinctly narrower than mentum.
25.	Mandibles (scored in females, if males have obviously modified mandibles): (0) symmetrical or nearly so (e.g. Antunes-Carvalho et al. 2017a: Fig. 3a, b); (1) left and right mandible differing in shape.
26.	Mesal mandibular tooth/teeth: (0) absent; (1) 1-2, situated on mesal margin (sometimes twisted ventrad or dorsad) near median coronal plane of mandible; (2) multiple (at least 3) teeth arranged in row on mesal margin near median coronal plane of mandible.
27.	Prostheca: (0) present (Fig. 4f); (1) absent (Yavorskaya et al. 2022: Fig. 2f).
28.	Mandibular mola: (0) absent (Yavorskaya et al. 2022: Fig. 2f); (1) present (e.g. Wheeler & Miller 2005: Fig. 16).
29.	Long patch or row of numerous setae along lateral or laterodorsal mandibular margin extending from base to at least middle of mandible, usually farther: (0) absent (a few sparse setae may be present) (Fig. 4e); (1) present (e.g. Antunes-Carvalho et al. 2017a: Fig. 3a).
30.	Long subapical or submedian seta on maxillary palpifer: (0) present; if other setae are present, they do not form brush and are not similar in length (one seta stands out as the longest) (e.g. Antunes-Carvalho et al. 2017a: Fig. 3d); (1) absent.
31.	Maxillary palpomere 2: (0) strongly elongate, clearly more than twice as long as broad; (1) short, less than twice as long as broad.
32.	Maxillary palpomere 3: (0) enlarged in relation to remaining palpomeres, not pedunculate, broadening distally, with nearly straight lateral margins (e.g. Antunes-Carvalho et al. 2017a: Fig. 3c); (1) not enlarged in relation to remaining palpomeres, subcylindrical or indistinctly clavate (Fig. 4j); (2) annulate.
33.	Maxillary palpomere 4: (0) subconical; (1) subuliform, fusiform or nearly cylindrical, strongly elongate, with rounded or truncate blunt apex.
34.	Setae on maxillary palpomere 4: (0) conspicuously dense, almost evenly distributed; (1) sparse, almost evenly distributed; (2) present and variously dense in basal half or 2/3, absent in distal region; (3) absent.
35.	Ventral field of dense slender sensilla forming fishbone pattern on maxillary palpomere 4: (0) absent; (1) present (Newton 1997: Fig. 9).
36.	Setal fringes of galea and lacinia: (0) composed mainly of large number of thin setae (usually clearly more than 30); (1) on galea mainly with thin setae, on lacinia mainly distinctly thickened, robust setae.
37.	Length of galea: (0) subequal to length of stipes; (1) much longer than stipes.
38.	Single lacinial apex: (0) inconspicuous, narrow and not particularly robust; (1) thick and claw-like, strongly sclerotized and robust.
39.	Mentum (sclerotized region): (0) subquadrate or transversely subrectangular; (1) trapezoidal.
40.	Lateral lobes of mentum projecting posteriorly: (0) absent; (1) present (Fig. 3b).
41.	Anterior margin of mentum: (0) straight or weakly arcuate; (1) distinctly but evenly and not conspicuously deeply concave.
42.	Insertions of labial palps: (0) separated by at most two diameters of palpomere 2; (1) separated by 3-4 diameters of palpomere 2.
43.	Labial palpomere 1: (0) nearly annulate, much broader than long; (1) 1-2 times as long as broad, not longer than 2; (2) 2-3 times as long as broad, shorter than 2; (3) elongate, distinctly longer than 2.
44.	Labial palpomere 2: (0) subcylindrical; (1) distinctly clavate.
45.	Antennal insertion: (0) anterolateral (not ventral), fully exposed in lateral view (but not in dorsal view) (Fig. 3a); (1) anterior or anteromedian and lateroventral, visible in lateral view; (2) anterior or anteromedian and dorsolateral, partly exposed in dorsal and lateral view; (3) anterior and almost entirely dorsal or dorsolateral, always fully exposed in dorsal view; (4) posterolateral, exposed in posterolateral view.
46.	Antenna: (0) composed of 11 antennomeres (Fig. 1a); (1) composed of 9 antennomeres (Yavorskaya et al. 2022: Fig. 3a).
47.	Antennomere 7: (0) not enlarged, at least narrower than 8 (Fig. 1a); (1) enlarged, longer, or broader, or longer and broader than 6 and 8 (e.g. Antunes-Carvalho et al. 2017a: Fig. 2f).
48.	Periarticular gutters on antennomeres 7, 9 and 10 or 8-10: (0) absent; (1) present (e.g. Antunes-Carvalho et al. 2017a: Fig. 2i).
49.	Form of periarticular gutters on distal antennomeres: (0) enclosed (e.g. Antunes-Carvalho et al. 2016: Fig. 2i); (1) open (Newton 1997: Fig. 3).
50.	Internal 'vesicles' inside antennomeres 7, 9 and 10 or 8-10: (0) present; (1) absent.
51.	Antennomere 10: (0) symmetrical or nearly so at least in females; (1) in both sexes asymmetrical, projecting laterally so that antennomere 11 appears as inserted on side of its apex.
52.	Cervical sclerites: (0) weakly sclerotized, poorly distinguishable from surrounding membrane; (1) strongly sclerotized, much darker than surrounding membranes, with cearly defined margins; (2) absent.
53.	Dorsolateral groove on pronotum to receive antenna: (0) absent; (1) present (Yavorskaya et al. 2022: Fig. 7a).
54.	Transverse ridges (strigae) densely covering pronotal disc: (0) absent; (1) present.
55.	Width of hypomeron in ventral view: (0) subequal to half width of furcasternum; (1) 1.5-2 times as broad as half width of furcasternum (Fig. 1c).
56.	Notosternal sutures: (0) present, complete or almost complete (Fig. 6a); (1) indiscernible or vestigial, marked only as barely discernible notches at anterior sternal margin (e.g. Wheeler & Miller 2005: Fig. 20).
57.	Precoxal region of prosternum: (0) much shorter than coxal rests (e.g. Wheeler & Miller 2005: Fig. 20); (1) much longer than coxal rests (Fig. 6a).
58.	Shape of anterior prosternal margin: (0) at middle not strongly projecting anteriorly; concave, straight or weakly, evenly convex; (1) at middle strongly projecting anteriorly.
59.	Procoxal cavities: (0) not separated or separated by weakly elevated longitudinal ridge hidden between procoxae (e.g. Antunes-Carvalho et al. 2017a: Fig. 8c); (1) separated by strongly elevated intercoxal process (Fig. 6a); (2) separated by weakly elevated but sharply delimited subtriangular process with rounded or pointed apex (Yavorskaya et al. 2022: Fig. 12h).
60.	Length of prosternum: (0) clearly less than half length of pronotum (e.g. Antunes-Carvalho et al. 2017: Fig. 8b, c); (1) as long as half-length of pronotum or longer (Fig. 6a).
61.	Relative width of exposed portion of mesoscutellar shield: (0) much narrower than 1/3 combined width of elytra (Fig. 1a); (1) subequal to 1/3 combined width of elytra (Yavorskaya et al. 2022: Fig. 1a); (2) nearly as broad as entire elytral base.
62.	Multiple setae on mesoscutellar shield: (0) absent; (1) present.
63.	Dense transverse ridges (=strigae) on elytra: (0) absent; (1) present (e.g. Antunes-Carvalho et al. 2017a: Fig. 12c).
64.	Elytral epipleurae: (0) inconspicuously narrow or virtually absent (Yavorskaya et al. 2022: Fig. 7b, f); (1) conspicuously broad, at least anteriorly (Fig. 10c).
65.	Placement of mesocoxal cavities: (0) distinctly closer to lateral margins of mesothorax than to its middle (e.g. Wheeler & Miller 2005: Fig. 21); (1) closer to middle than to lateral margins of mesoventrite (Fig. 6a); (2) approximately at middle between lateral margin and midline of mesoventrite.
66.	Protrochantin: (0) exposed (Fig. 6a); (1) concealed (Yavorskaya et al. 2022: Fig. 7c).
67.	Mesotrochantin: (0) exposed (Fig. 6a); (1) concealed (e.g. Æurèiæ et al. 2023: Fig. 2i).
68.	Placement of mesoventral process: (0) mostly between mesocoxae (Fig. 6a); (1) mostly between and/or in front of mesocoxal bases.
69.	Shape of mesoventral process between mesocoxae: (0) carinate or subcarinate (Yavorskaya et al. 2022: Fig. 12i); (1) subtriangular, distinctly narrowing posterad (e.g. Æurèiæ et al. 2023: Fig. 2i); (2) subtrapezoidal, broadest anteriorly, or broadly subrectangular (Fig. 6a).
70.	Subtriangular mesoventral process: (0) strongly elongate; (1) about as long as broad; (2) clearly broader than long.
71.	Subtrapezoidal and broadest anteriorly mesoventral process: (0) 1.5-2 times as long as broad; (1) strongly elongate, 3-5 times as long as broad; (2) strongly transverse.
72.	Mesoventral process in lateral view: (0) in front of mesocoxae weakly elevated or absent; (1) in front of mesocoxae distinctly elevated.
73.	Sides of mesoventral process: (0) not expanding laterally to overhang mesal coxal margins (Yavorskaya et al. 2022: Fig. 12i); (1) ventral surface expanded laterally and enclosing mesal regions of mesocoxae (process inversely T-shaped in cross-section) (Fig. 6a).
74.	Posterior margin of mesoventral intercoxal process: (0) fused with metaventrite (Yavorskaya et al. 2022: Fig. 12i); (1) clearly separated from metaventrite (Fig. 6a).
75.	Anterior metaventral process: (0) absent (Fig. 6a); (1) broad, subtrapezoidal with truncate anterior margin or subquadrate; (2) narrow, subrectangular, subtrapezoidal, subtriangular or anteriorly rounded; (3) broad, subtriangular, with narrow and pointed or narrowly rounded apex.
76.	Metanepisterna: (0) demarcated from metaventrite (e.g. Wheeler & Miller 2005: Fig. 21); (1) fused with metaventrite.
77.	Posterior admetacoxal margin of metaventrite: (0) arcuate or weakly sinuate; (1) straight or nearly so.
78.	Deep and narrow median notch or emargination in posterior margin of intermetacoxal katepisternum: (0) absent; (1) present.
79.	Paracoxal sutures on metaventrite: (0) absent; (1) clearly marked on surface.
80.	Metendosternite: (0) Y-shaped with narrow and at least slightly elongate stalk (Fig. 5b); (1) T-shaped, with distal region transverse; (2) cross-shaped, with transverse lateral arms distant from apex of median projection, which is nearly as broad as stalk.
81.	Stalk in Y-, T- or cross-shaped metendosternite: (0) at most 3 times as long as broad (Fig. 5b); (1) slender, 5 and more time as long as broad.
82.	Metascutum: (0) laterally not overlapping meso- and metapleural regions; (1) laterally overlapping meso- and metapleural regions.
83.	Exposed portion of procoxa: (0) subglobose or only slightly elongate (Fig. 6b); (1) subconical, broadest proximally, strongly projecting ventrally.
84.	Length of procoxa: (0) at most as long as half-length of profemur (Fig. 6b); (1) conspicuously large, longer than half length of profemur.
85.	Mesocoxa: (0) oval; (1) subconical, broadest in basal half and elongate; (2) subglobose (Fig. 6a).
86.	Length of mesocoxa: (0) conspicuously long, much longer than half length of mesofemur; (1) small, at most as long as half width of mesofemur (Fig. 6f).
87.	Shape of metacoxa: (0) in ventral view subtriangular; (1) in ventral view subrectangular or oval (Fig. 6a).
88.	Adfemoral articulating margin of mesotrochanters (and often also protrochanters): (0) distinctly shorter than basal articulating margin of femur, so that posterodorsal region of femoral base is protruding mesally over trochanter (Yavorskaya et al. 2022: Fig. 12i); (1) as long as or slightly longer than basal articulating margin of femur.
89.	Sparse robust spines unevenly distributed on entire length of at least mesotibiae (often in all tibiae): (0) absent; (1) present (Fig. 6e, f).
90.	Serrate (or at least tricuspidate) spurs on apices of all tibiae: (0) absent; (1) present (e.g. Antunes-Carvalho et al. 2017: Fig. 15i).
91.	Tarsal formula: (0) same in males and females; (1) different in males and females.
92.	Tarsal formula same in males and females: (0) 5-5-5; (1) 5-5-4; (2) 5-4-4.
93.	Tarsal formula different in males and females: (0) in males 5-5-4, in females 5-4-4; (1) in males 5-5-5, in females 4-5-5; (2) in males 5-5-4, in females 4-4-4.
94.	Protarsi in females: (0) distinctly flattened and broadened; (1) not flattened and broadened.
95.	Protarsi in males: (0) not broadened and flattened; (1) distinctly broadened and flattened.
96.	Intersegmental abdominal membranes: (0) lacking dense and regular brick-wall pattern of minute sclerites smooth; (1) with dense and regular brick-wall pattern of minute sclerites.
97.	Tergites exposed when abdomen is fully stretched: (0) none or only tip of last visible; (1) more than one (Fig. 1a).
98.	Spiculum ventrale (median proximal projection) of female sternite VIII: (0) absent; (1) present.
99.	Gonostylus: (0) present; (1) absent.
100.	Spermatheca: (0) sclerotized; (1) unsclerotized.
101.	Aedeagal orifice (= basal foramen) [and parameral bases, if present]: (0) on ventral or dorsal surface and in proximal half of median lobe; (1) on basal surface of median lobe (scored as basal even if median lobe is curved).
102.	Parameres: (0) present; (1) absent.
103.	Annulate phallobase (= basal piece) embracing median lobe of aedeagus, on which parameres are inserted: (0) absent; (1) present.
104.	Arcuate basal piece at base of parameral insertion, below orifice of aedeagus: (0) absent; (1) present.


References (not in the maintext) 

Æurèiæ, S., Vesoviæ, N., Vribica, M., Popoviæ, S., Rodanoviæ, Ž., Æurèiæ, N.B., Yamashkin, A.A., Radoviæ, D., Tamashkin, S.A., Vraniæ, S., Raða, T. The surprising discovery of two new subterranean Leptodirini of the genus Spelaeobates Müller, 1901 (Coleoptera, Leiodidae, Cholevinae) from Croatia after more than a century. Subterranean Biology, 46, 21-46.
Newton, A.F. (1997) Review of Agyrtidae (Coleoptera), with a new genus and species from New Zealand. Annales Zoologici, 47(1/2), 111-156.
Wheler, Q.D., Miller, K.B. (2005) Slime-mold beetles of the genus Agathidium Panzer in North and Central America, part I. Coleoptera: Leiodidae. Bulletin of the American Museum of Natural History, 290, 1-95.
Zhou, H.-Z., Yu, X.-D., Luo, T.-H. (2005) Discovery of the subfamily Necrophilinae (Coleoptera: Agyrtidae) in China and description of a new species of Necrophilus Latreille. The Coleopterists Bulletin, 59(1), 62-69.
